# Surface chemical defence of the eelgrass *Zostera marina* against microbial foulers

**DOI:** 10.1038/s41598-019-39212-3

**Published:** 2019-02-26

**Authors:** Stefano Papazian, Delphine Parrot, Barbora Burýšková, Florian Weinberger, Deniz Tasdemir

**Affiliations:** 1 GEOMAR Centre for Marine Biotechnology, Research Unit Marine Natural Products Chemistry, GEOMAR Helmholtz Centre for Ocean Research Kiel, Am Kiel Kanal 44, 24106 Kiel, Germany; 2 Research Unit Marine Benthic Ecology, GEOMAR Helmholtz Centre for Ocean Research Kiel, Düsternbrooker Weg 20, 24105 Kiel, Germany; 3 Kiel University, Christian-Albrechts-Platz 4, 24118 Kiel, Germany

## Abstract

Plants rely on both mechanical and chemical defence mechanisms to protect their surfaces against microorganisms. The recently completed genome of the eelgrass *Zostera marina*, a marine angiosperm with fundamental importance for coastal ecosystems, showed that its re-adaptation from land to the sea has led to the loss of essential genes (for chemical communication and defence) and structural features (stomata and thick cuticle) that are typical of terrestrial plants. This study was designed to understand the molecular nature of surface protection and fouling-control strategy of eelgrass against marine epiphytic yeasts. Different surface extraction methods and comparative metabolomics by tandem mass spectrometry (LC-MS/MS) were used for targeted and untargeted identification of the metabolite profiles of the leaf surface and the whole tissue extracts. Desorption electrospray ionization-imaging mass spectrometry (DESI-IMS) coupled with traditional bioassays revealed, for the first time, the unique spatial distribution of the eelgrass surface-associated phenolics and fatty acids, as well as their differential bioactivity against the growth and settlement of epiphytic yeasts. This study provides insights into the complex chemical defence system of the eelgrass leaf surface. It suggests that surface-associated metabolites modulate biotic interactions and provide chemical defence and structural protection to eelgrass in its marine environment.

## Introduction

Seagrass meadows are widespread along the coastlines of the world oceans, where they prevent sediment erosion and provide numerous animal species with food and shelter, thus supporting the stability of marine habitats^1^. As primary producers, seagrasses influence the dynamics of biogeochemical processes, such as carbon sequestration, oxygen production and organic matter deposition^2–4^. The eelgrass *Zostera marina* L. (Zosteraceae) is one of the most successful marine angiosperms and at the same time one of the best-studied seagrass models. The recently completed genome sequencing of *Z*. *marina* showed that its re-adaptation from land to sea approximately 150 million years ago required regaining of genes for salinity tolerance, e.g. production of osmolytes and cell wall analogues of those found in macroalgae, but also led to the loss of genes coding for structural and physiological features typical of land plants, e.g. thick leaf cuticles, stomata, terpene synthesis, ethylene signaling and UV protection^5,6^. These findings raise the question whether eelgrass and seagrasses in general still retain the complexity and characteristics of angiosperms for interspecies communication and chemical protection.

Interactions between macrophytes and marine microorganisms are largely defined at the host surface^7^. The epibionts and pathogens that constantly colonize the leaf surfaces of eelgrass can hamper its growth at initial developmental stages^8^. In addition, microbial foulers affect eelgrass stress response capability by limiting its access to light, oxygen and nutrients^9^, and can potentially modulate the composition of the total surface-associated microbiome^10^. This interaction between biotic and abiotic factors can have detrimental effects on the physiological state, the natural defence system and ultimately the fitness of *Z*. *marina*^11,12^. For instance, during the 1930s and 1980s in the Northern Atlantic, the high susceptibility of eelgrass to the parasitic slime mold *Labyrinthula zosterae* resulted in large epidemics of the ‘wasting disease’ and massive die-offs^11,13^. Remarkably, under normal healthy circumstances, seagrasses including *Z*. *marina* do not suffer from extended epibiosis or fouling^7,14,15^, suggesting the presence of innate defensive mechanisms possibly mediated by surface-associated metabolites^16^. The phenolic compounds isolated from the whole leaf tissue extracts of *Z*. *marina*, e.g. rosmarinic acid (RA), zosteric acid (ZA), and sulfated flavonoids are known to act as antimicrobial or antifouling agents^17–21^. However, in order to be involved in chemical signaling and fight epibiosis, these allelochemicals would require to be produced intracellularly and then to be released into the first site of contact with the microfoulers, i.e. the leaf surface^16,19–23^. Yet, little is known about their actual surface distribution in *Z*. *marina*.

Only few methods are available for extraction of plant surfaces. These include the classical ‘surface dipping’ method, which involves the brief immersion of the leaves in organic solvents^19^, or the solid-phase extraction method, recently developed for studying seaweed surfaces, in which chemicals are first adsorbed onto C18 material and then eluted with a solvent^24^. Both methods are tedious and entail limitations, e.g. poor recovery or reproducibility, potential degradation of the compounds, and the risk of physical or chemical damage to the tissues, which result in the co-extraction of metabolites from the epidermis. Most importantly, these methods cannot provide any information on the localization or spatial distribution of surface metabolites, which is important to understand their ecological functions.

Conventional mass spectrometry (MS) has become an indispensable tool in natural product research and metabolomics, combining fast chemical screening with extreme sensitivity and wide dynamic range. Automated workflows, such as dereplication by molecular networking (MN) that uses MS/MS fragment similarity for the prediction and clustering of molecular species^25,26^, are becoming established approaches in high-throughput metabolomics. Imaging mass spectrometry (IMS) has the ability to further extend the power of MS by simultaneously providing the chemical and spatial information of biological samples at the μm-scale resolution^27^. Desorption electrospray ionization-imaging mass spectrometry (DESI-IMS) employs a continuous flow of charged solvent droplets for the soft ionization of the analyte surfaces at ambient conditions^27,28^. Thus, DESI-IMS is a versatile tool in chemical ecology for spatial analysis of biological surfaces and for the identification of host-microbe interactions, as demonstrated by pioneering studies on seaweeds and corals^29,30^.

In a previous study, we identified RA and several sulfated flavonoids from surface solvent extracts of the Baltic *Z*. *marina* as its active components against marine fouling microorganisms^19^. Herein, we applied a comparative LC-MS metabolomics approach for analysing the surface, the whole leaf-, and the surface-free (i.e. whole leaf after surface dipping) extracts of the eelgrass, combined with the chemical imaging of the leaf surface imprints by DESI-IMS. Bioactivity of the extracts and their major constituents was assessed on two microfouling yeasts of *Z*. *marina -* namely the facilitator of the wasting disease *Cryptococcus fonsecae* and the halophyte *Debaryomyces hansenii* (syn: *Candida famata*), which is known to confer host-antagonist-pathogen protection in terrestrial plants inducing phytoalexin biosynthesis^31,32^. Our results provide evidence for a selective chemical defence system in eelgrass involving surface-associated phenolics and fatty acids (FAs) to control growth and settlement of marine fouling microorganisms.

## Results

### Antimicrobial activity of eelgrass leaf surface and whole tissue extracts

**Figure 1:**
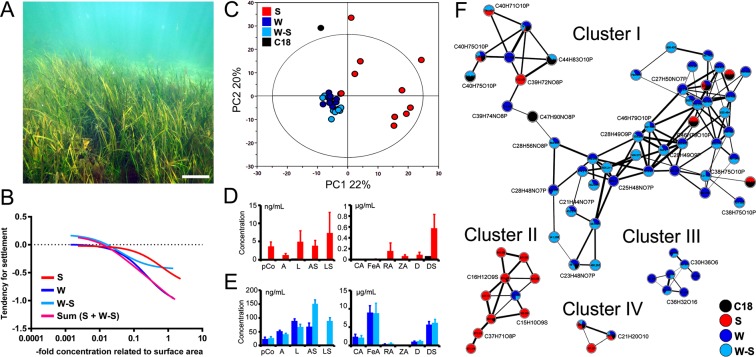
Leaf tissue and surface-associated chemistry of *Z*. *marina* involved in antifouling defence. (**A**) Eelgrass meadow in the Baltic Sea, Kiel Fjord. Scale bar = approx. 30 cm. Photo by Stefano Papazian. (**B**) Cumulative regression model (Sum, purple) comparing the inhibitory antifouling activity of eelgrass extracts obtained by surface dipping extraction (S, red), whole leaf (blue, W), and whole leaf after surface dipping (light-blue, W-S), on the settlement of the marine epiphytic yeast, *D*. *hansenii*. (**C**) Multivariate analysis (PCA 3 components) for comparative LC-MS/MS metabolomics of surface solid-phase (C18), solvent dipping (S), whole leaf (W) and surface-free (W-S) extracts. (**D,E**) Concentrations of phenolic metabolites detected in the (**D**) surface extracts (S, C18) and (**E**) whole leaf extracts (W, W-S), i.e. *p*-coumaric acid (*p*-Co), apigenin (A), luteolin (L), apigenin-7-sulfate (AS), luteolin-7-sulfate (LS), caffeic acid (CA), ferulic acid (FeA), rosmarinic acid (RA), zosteric acid (ZA), diosmetin (D), and diosmetin-7-sulfate (DS). Error bars = standard error. (**F**) Molecular network built in GNPS from spectral data obtained from LC-MS/MS analyses of all extracts. Four main clusters show the dereplication and respective chemical groups annotated as phenolics, phospholipids, and fatty acid esters (Supplementary Table S2).

All eelgrass extracts, i.e. the solid-phase adsorption (C18), solvent dipping (S), whole leaf (W), and surface-free (W-S) extracts, were tested against two marine yeasts, *C*. *fonsecae* and *D*. *hansenii*. Both the C18 and the S surface extracts, but not the W and W-S extracts, inhibited the growth of *C*. *fonsecae* (25–40%) at concentration >1 µg.cm^−2^, but showed no effect on the growth of *D*. *hansenii* (Supplementary Figs S22, S23 and Supplementary Tables S7, S8). Except for the C18, all extracts (S, W, and W-S) negatively affected the settlement of *D*. *hansenii* (60–90%) at concentrations >0.01 µg.cm^−2^ (Supplementary Fig. S24 and Supplementary Table S9). When dose responses of the surface and whole leaf tissue extracts were related to 1-fold concentrations found in *Z*. *marina*^19^, all models (except the C18) showed selective inhibition against either growth or settlement of the yeasts (Supplementary Figs S22–S24). Cumulative linear regression models showed the additive antisettlement effect of the surface (S) and surface-free (W-S) extracts against *D*. *hansenii*, resulting in a perfect fit to the individual model obtained from the whole leaf extracts (W) (Fig. 1B).

### Identification of sulfated phenolics as main metabolites in the leaf surface and whole tissue extracts

The LC-MS profiles of all eelgrass extracts (Fig. 1C,D and Supplementary Fig. S4) showed clear differences. Based on the multivariate principle component analysis (PCA; Fig. 1C and Supplementary Table S4), the largest deviation was displayed by the C18 extract followed by the S extracts with high variability (including two replicates outside the Hotelling’s *T*^2^ distribution), whereas the whole leaf (W) or surface-free (W-S) extracts had highly uniform profiles. Twelve phenolic metabolites (Table 1), i.e. RA, *p*-coumaric acid (*p*-Co), caffeic acid (CA), ferulic acid (FeA), zosteric acid (ZA), three sulfated flavonoids apigenin-7-sulfate (AS), luteolin-7-sulfate (LS), diosmetin-7-sulfate (DS) and their respective desulfated forms (A, L, and D), were identified as the major constituents of eelgrass leaf surfaces and whole tissues. Also kaempferol-7,4′-dimethylether-3-O-sulfate was putatively identified (Table 1 and Supplementary Figs S4, S8–S13). The disaccharide trehalose was found to be more abundant on the leaf surface (S) than in the whole tissues (W and W-S) (Supplementary Figs S4 and S14). These results were further supported by a supervised discriminant analysis for the same extracts showing the respective metabolite contribution (PLS-DA Supplementary Fig. S7 and Table S5).

**Table 1:** Metabolites identified in the eelgrass leaf tissue- and surface extracts.

Formula	Metabolite ID	*m/z* [M-H]^−^	RT (min)	MS/MS	Ref.	Score	LC-MS	DESI-IMS
C6H12O3	3-Hydroxyhexanoic acid	131.071	—	—	—	*		X
C9H8O3	***p*** **-Coumaric acid**	163.039	3.6	—	^34, 37^	**		X
C9H16O3	4-Hydroxynonenoic acid	171.102	—	—	—	*		X
C9H8O4	**Caffeic acid**	179.034	3.3	135	^37, 38^	***	X	X
C9H16O4	**Azelaic acid**	187.097	—	—	—	**		X
C10H10O4	**Ferulic acid**	193.049	4.3	178, 134	^63^	***	X	
C14H28O2	**Myristic acid**	227.201	—	—	^21^	**		X
C9H8O6S	Zosteric acid	242.995	3.1	—	^19, 37^	***	X	X
C16H30O2	**Palmitoleic acid**	253.218	—	—	^21^	**		X
C16H32O2	**Palmitic acid**	255.232	—	—	^21^	**		X
C15H10O5	**Apigenin**	269.045	4.3	—	^17, 37^	***	X	X
C18H32O2	Linoleic acid	279.232	—	—	^21^	**		X
C18H34O2	**Oleic acid**	281.248	—	—	^64, 65^	**		X
C15H10O6	**Luteolin**	285.031	4.2	243, 83	^17, 19, 37^	***	X	X
C16H12O6	**Diosmetin**	299.053	4.5	284, 256	^17, 19, 37^	***	X	X
C15H10O8S	Apigenin-7-sulfate	349.003	4.4	269	^37^	***	X	X
C18H16O8	**Rosmarinic acid**	359.078	4.1	—	^19, 37, 38^	***	X	X
C15H10O9S	Luteolin-7-sulfate	364.996	4.2	285	^17, 19, 37^	***	X	X
C16H12O9S	Diosmetin-7-sulfate	379.012	4.5	299, 284	^17, 19, 37^	***	X	X
C12H22O11	Trehalose (+FA adduct)	387.114	0.6	341, 179, 161	^5^	***	X	
C17H14O9S	Kaempferol-7,4′-dimethylether-3-O-sulfate	393.029	4.9	351, 325, 313	—	**	X	

Compounds identified in the *Z*. *marina* leaf surface (S) and the whole leaf extracts without or with previous surface dipping (W and W-S) detected by UHPLC-QTOF-MS and/or by DESI-IMS on leaf surface imprints. Metabolites were annotated by comparison of their negative mode MS spectra with reference literature and/or METLIN within a 1–10 Δ ppm mass tolerance, and by consideration of *in silico* or experimental MS/MS fragmentation data when available. A relative score was assigned as certain (***) or high confidence (**) identification for compounds previously reported in *Zostera* sp., seagrasses, and/or with supportive MS/MS spectra, or (*) for putative identifications. Compounds detected in extracts by UHPLC-QTOF-MS (Fig. S4) and/or on leaf surfaces by DESI-IMS (Figs S16–20) are marked with X. Compounds highlighted in bold were tested for their activity in bioassays against microfoulers (Figs S25–28). FA: formic acid.

The concentrations of the phenolic compounds in the extracts were quantified by comparison with pure standards (Fig. 1D,E and Supplementary Fig. S15). The most abundant phenolic detected in the surface extract (S) was diosmetin-7-sulfate (DS), with 10-fold higher concentrations (0.57 µg.ml^−1^) than the C18 extract (0.06 µg.ml^−1^). Its desulfated form D had low (0.09 µg.ml^−1^) or trace amounts in the S and C18 extracts, respectively. The next most abundant phenolic in the S extract (absent in the C18) was ZA (0.07 µg.ml^−1^), followed by lower concentrations of CA, FA, *p*-Co, and the sulfated flavonoids LS and AS. Notably, RA had only trace levels in S extracts (<0.60 ng.ml^−1^), except for two replicates with high levels of RA (1.3–52.6 µg.ml^−1^) corresponding to the two outliers highlighted in the PCA model (Fig. 1C; see also Supplementary Fig. S5 and Supplementary Table S1). The W and W-S extracts were rich in FeA and CA (8.95 and 2.38 µg.ml^−1^). Except for ZA, all the other phenolics were detected in W and W-S at 10-fold higher concentrations than in the S extracts (Fig. 1D,E and Supplementary Table S1).

Based on the spectral fragmentation data obtained from untargeted LC-MS/MS analyses, mass features of all extracts were characterized using automated MN. Nodes representing related molecular clusters (I-IV) were automatically grouped and annotated with matches from the GNPS libraries (https://gnps.ucsd.edu) (Fig. 1F and Supplementary Table S2). The whole tissue (W and W-S) and the surface (C18 and S) extracts were largely represented in the first (I) and second (II) largest molecular clusters (*m/z* [M-H]^−^ 407–826) by the chemical families glycerophosphoglycerol, phosphatidylcholine, glycerophosphoethanolamine, phosphatidic acid and the esters of FAs (including linolenic, oleic, and palmitic acid). Additionally, two nodes from the surface extracts (C18, S) in the second cluster (II) that did not match the GNPS libraries were manually annotated by MS/MS spectra dereplication as the sulfated flavonoids DS and LS (*m/z* [M-H]^−^ 379 and 365). The remaining two smaller clusters (III, IV) matched the molecular families of phenolic compounds such as sophoricoside (*m/z* [M-H-CO_2_]^−^ 387), sophoraisoflavanone D (*m/z* [M-H]^−^ 491), and sagerinic acid (*m/z* [M-H-CH_2_O_2_]^−^ 671) (Fig. 1F and Supplementary Table S2). Additional mining for unknown compounds was performed by computing MS/MS spectral fragmentation trees in SIRIUS, which suggests candidate molecular formula based on *in-silico* predictions (Supplementary Table S3).

### Differential distribution of sulfated phenolics and fatty acids on the eelgrass surface by DESI-IMS

**Figure 2:**
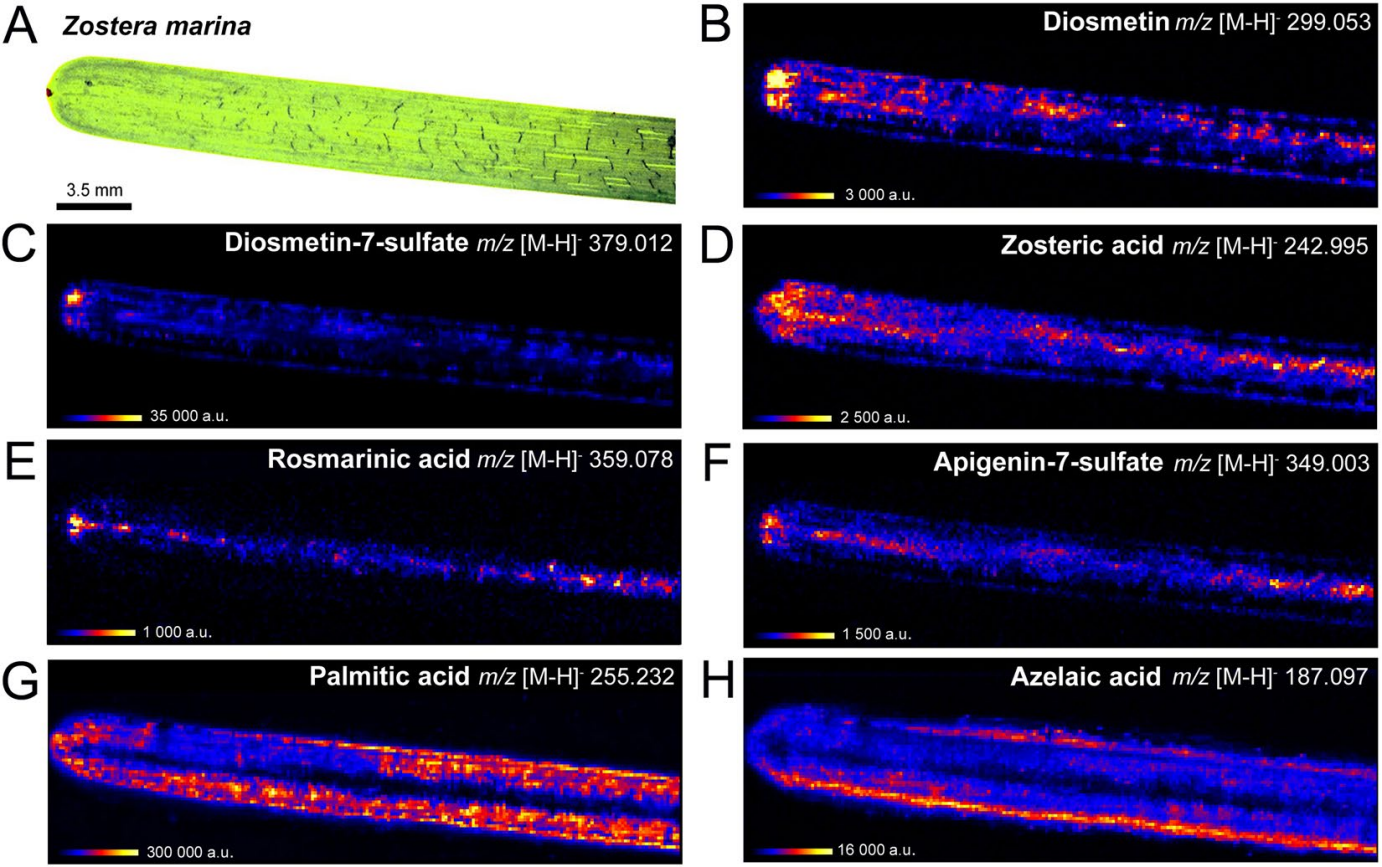
Spatial distribution of the eelgrass surface metabolites by DESI-IMS. Photograph of eelgrass *Z*. *marina* leaf-blade, scale bar = 3.5 mm (**A**), and DESI-IMS images at 150-μm lateral resolution showing distribution and relative intensity of *m/z* [M-H]^−^ ions for the metabolites (**B**) diosmetin, (**C**) diosmetin-7-sulfate, (**D**) zosteric acid, (**E**) rosmarinic acid, (**F**) apigenin-7-sulfate, (**G**) palmitic acid, and (**H**) azelaic acid. Heat-map scaling shows the highest local accumulation points indicated by the respective maximum range on the intensity scale (a.u.). Total scanned surface area = 312 mm^2^ (3.1 cm^2^). Actual scanned leaf surface = 125 mm^2^ (1.25 cm^2^).

**Figure 3:**
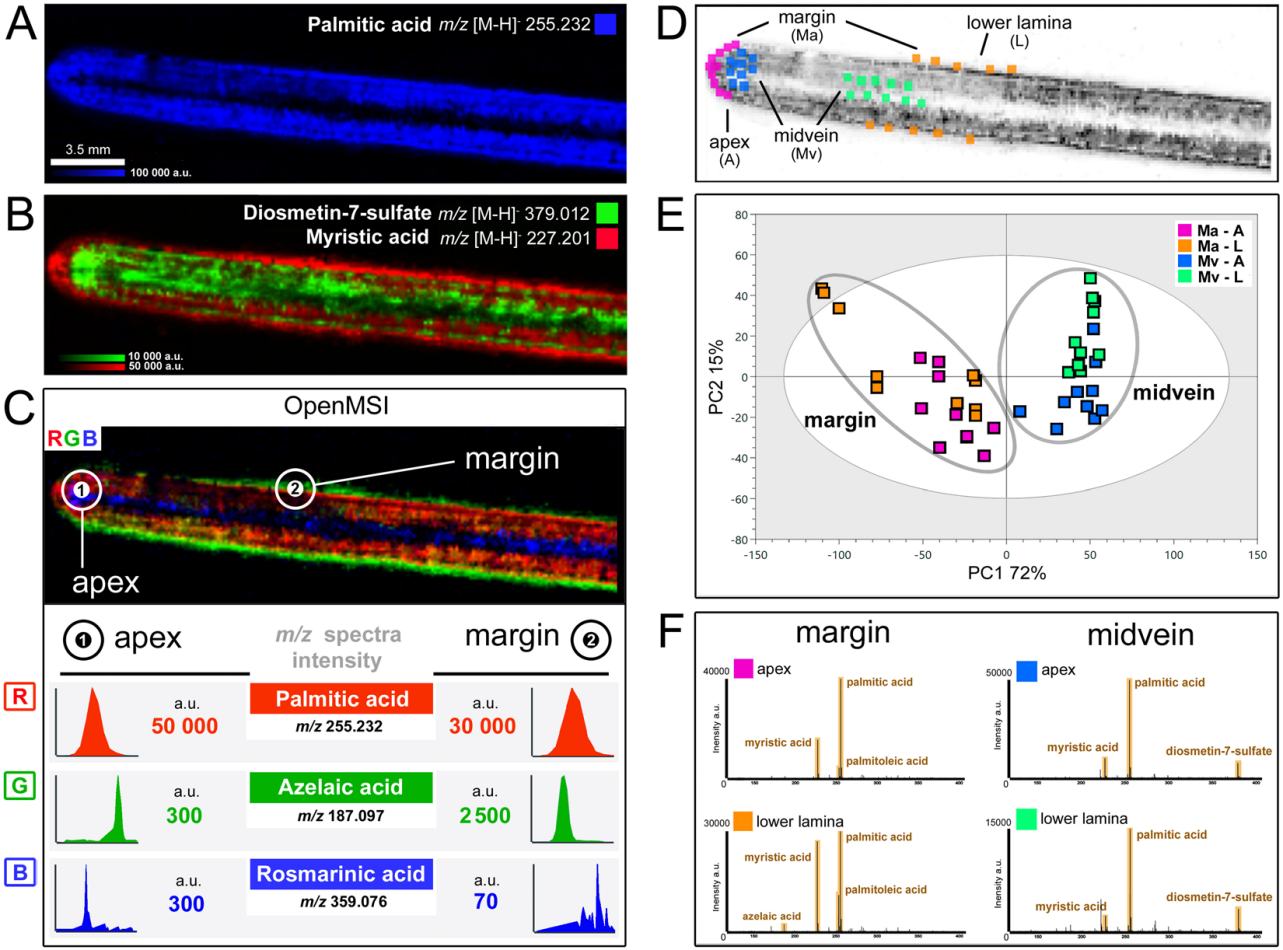
Specific localization of *Z*. *marina* surface metabolites. DESI-IMS showing the distribution on the eelgrass leaf of (**A**) palmitic acid, and (**B**) superimposition of myristic acid (red) and diosmetin-7-sulfate (green). (**C**) Analysis in OpenMSI (https://openmsi.nersc.gov) showing metabolite distribution via RGB-color superimposition of palmitic acid (red), azelaic acid (green), and rosmarinic acid (blue), and respective intensity of *m/z* ions in the DESI-IMS spectra at the apex (1) and at the margin (2). Images are scaled to the average intensity for each ion (no cut-off). (**D**) The DESI-IMS spatial-chemical information across four regions of interest (ROI) of the leaf surface, i.e. apex, lower lamina, margin, and midvein (10-sample points), were modelled by chemometrics, with (**E**) PCA showing ROI cluster intra- and inter-cluster metabolic variation, and (**F**) respective spectral similarity in OpenMSI.

In order to analyse the surface-associated metabolites on eelgrass leaves while avoiding tissue damage or cell disruption^27^, DESI-IMS was performed on eelgrass surface imprints obtained on clean glass slides (Supplementary Figs S16–S20). DESI-IMS screening was performed at 150-μm resolution across the 1.25 cm^2^ leaf surface area (Fig. 2A–H). Sequential processing and visualization of the multi-dimensional *m/z* data showed the distribution and relative intensity of *m/z* [M-H]^−^ ions of different phenolic compounds on the leaf surface (Fig. 2B–F and Supplementary Fig. S17). Consistent with LC-MS results (Fig. 1D), the most abundant phenolic compound detected by DESI-IMS was DS (Fig. 2C), while lower levels were detected for the sulfated flavonoids LS and AS and the phenolic acids ZA and RA (Fig. 2D–F and Supplementary Fig. S17). Compared to surface average abundances, concentration maxima of the phenolic compounds were estimated at 0.2 µmol.cm^−2^ for DS and ZA (Supplementary Fig. S21) and in the lower range of 0.1 µmol to 1 nmol.cm^−2^ for LS, AS and RA (Supplementary Table S1). Only traces of *p*-Co and CA were detected by DESI-IMS on the surface (Supplementary Fig. S17), while FeA was absent. DESI-IMS further revealed the presence of other metabolites on the surface (Fig. 3G,H and Table 1, Supplementary Fig. S18) including three linear carboxylic acids, 3-hydroxyhexanoic acid, 4-hydroxynonenoic acid and nonanedioic acid - also known as azelaic acid (Supplementary Fig. S18) - and five FAs, palmitic, palmitoleic, myristic, linoleic and oleic acids (Supplementary Figs S18). Particularly, palmitic acid (C16) and myristic acid (C14) (Fig. 3A,B) were  detected as the most abundant surface-associated metabolites with concentrations estimated to be approx. 5 to 10-fold higher than DS (Fig. 3B and Supplementary Fig. S18).

DESI-IMS showed the highly diverse distribution pattern of phenolics and FAs across the leaf-blade. The major flavonoid DS (and desulfated D) displayed a patchy but central distribution along the midvein and the two lateral veins, with highest levels at the apex (Fig. 2B,C and Supplementary Fig. S17). A similar localization along the midvein and lateral veins with highest accumulation at the apex was also shown at different degrees by ZA, LS, AS, and RA (Fig. 2D–F and Supplementary Fig. S17). On the contrary, the linear carboxylic acids and FAs were localized around the leaf margins (Supplementary Fig. S18). The only exception was palmitic acid, which was uniformly distributed over the entire leaf-blade surface albeit with lower intensity along the midvein (Fig. 2G and Supplementary Fig. S18).

We used the dedicated platform for IMS data analysis OpenMSI^33^ to further investigate the distribution of these phenolics and FAs by simultaneously visualizing the DESI-IMS images and spectra and pin-point scanning *m/z* ions throughout multiple surface locations (Fig. 3C). The chemical variation captured by DESI-IMS on the eelgrass surface was quantified by chemometric modelling of the spatial profile between four regions of interest (ROI; 10 sample points) randomly selected around the apex and the lower lamina, and along the margin and the midvein (Fig. 3D). A PCA of the multi-dimensional profiles highlighted the chemical heterogeneity of the eelgrass leaf surface (Fig. 3E). Compared to the midvein regions, a higher metabolic variation was displayed around the leaf margins particularly towards the lower lamina (with two sample points clustered outside the Hotelling’s *T*^2^ distribution). Overall, the variation between the margin and the midvein regions was greater than that between the apex and the lower lamina, as described by the first (PC1, 72%) and second (PC2, 15%) model components, respectively (Fig. 3E and Supplementary Table S6).

Targeted spectral analysis in OpenMSI for each of the ROIs confirmed the strongest difference between the margin and midvein surface regions within the lower lamina and the apex (Fig. 3F). Particularly, the sulfated flavonoid DS displayed the highest accumulation along the midvein and around the apex, with up to ~200-fold higher intensity compared to the leaf margin (Fig. 3F and Supplementary Figs S17 and S21). Instead, the major ion in the DESI-IMS spectra, i.e. palmitic acid, displayed high and homogenous intensity within the margin and midvein regions near the apex, but was much less abundant along the midvein near the lower lamina (Fig. 3F and Supplementary Fig. S18). Other FAs, e.g. myristic and palmitoleic acids, and similarly azelaic acid, showed a highly specific distribution around the margin, with up to 10-fold higher intensities towards the lower lamina than other regions (Fig. 3F and Supplementary Fig. S18). Moreover, compounds such as 3-hydroxyhexanoic acid, as well as oleic and myristic acids, showed a high accumulation within a small region of the leaf margin surrounding the apex (Supplementary Figs S18 and S19).

### Specific antimicrobial activity of the eelgrass surface-associated metabolites

Next, we investigated the potential of the surface-associated metabolites to inhibit the growth and fouling of *C*. *fonsecae* and *D*. *hansenii*. Due to the instability of the sulfate group of flavonoids and phenolic acids contained in seagrasses including *Zostera* sp^18^. these compounds are difficult to be purified directly from the plant in concentrations that are suitable for bioassays. To overcome this limitation, bioassays in this study were performed using the commercially available desulfated flavonoids (D, L, A), and instead of ZA we tested *p*-Co, which is known to be released *in vivo* by the sulfatase enzyme and considered as the bioactive form^34^. No inhibition was observed on the growth of the yeasts by RA or the flavonoids (Supplementary Figs S25, S26 and S28). Instead, all flavonoids inhibited the settlement of *D*. *hansenii* (75–100%) at concentrations >0.1–1 nmol.cm^−2^ (Supplementary Figs S27 and S28 and Supplementary Table S9). *p*-Co inhibited the growth of *C*. *fonsecae* (80%) as well as the growth and the settlement of *D*. *hansenii* (50–100%) at concentrations >0.1 µmol.cm^−2^ (Supplementary Figs S25 and S28 and Supplementary Tables S7–S9). Different degrees of bioactivity were observed for the carboxylic acids and FAs. At concentrations >0.1 µmol.cm^−2^, palmitic acid inhibited the growth of *C*. *fonsecae* (98%) but facilitated the settlement of *D*. *hansenii* (150%), whereas compounds that were specifically distributed at the margins cumulatively inhibited all the yeast models (50–100%), i.e. growth of *C*. *fonsecae* (myristic acid), settlement of *D*. *hansenii* (palmitoleic acid), and both settlement and growth for each yeast (azelaic acid) (Supplementary Figs S25–S27 and Supplementary Tables S7–S9). Similarly, at concentrations >0.1 µmol.cm^−2^, oleic acid slightly promoted the growth of *C*. *fonsecae* (35%) but did not affect either growth or settlement of *D*. *hansenii* (Supplementary Figs S25–S27).

## Discussion

Analysis of the eelgrass leaf chemistry by surface extraction methods provided different yields and chemical profiles (Fig. 1). The solid-phase (C18) adsorption method appeared inefficient for *Z*. *marina*, possibly due to low adhesion of the material on the smooth leaf surfaces. Solvent dipping (S) was simpler, quicker and more effective, but suffered from higher variability compared to the leaf tissue extractions (W, W-S) that were prepared by using the automated Accelerated Solvent Extraction (ASE) system. Extraction recovery in surface-free extracts (W-S) was occasionally more efficient compared to W extracts, possibly due to partial removal of the thin cuticle layer of *Z*. *marina*^35,36^. When tested against the marine yeast models, only the surface extracts (C18 and S) inhibited the growth of *C*. *fonsecae*, whereas all extracts (except the C18) inhibited the settlement (but not growth) of *D*. *hansenii*. Notably, the activity of the whole leaf extracts (W) versus the settlement of *D*. *hansenii* was similar to that of surface (S) and surface-free (W-S) extracts combined (Fig. 1B), supporting the contribution of surface-associated metabolites in the protection of eelgrass against microfoulers.

The major constituent of the eelgrass leaf surfaces, as detected by LC-MS in both C18 and S extracts, was the sulfated flavonoid DS (and its desulfated form D). ZA was the second most abundant phenolic compound detected in S, followed by lower levels of the sulfated flavonoids AS and LS. Possibly due to technical variation inherent to the solvent dipping method, high surface levels of RA were detected only in two S replicates (Supplementary Table S1). Chemical variability can also result from biological variation of individual chemotypes^37,38^ causing deviations between replicates, such as those with higher levels of RA (Supplementary Fig. S5 and Supplementary Table S1). Geographical and temporal variations also affect the leaf chemical profiles^37,38^. For example, our previous study^19^ detected high levels of RA in the surface extracts of the eelgrass collected from the same location in March (2013) compared to the specimens from early September (2017) used herein. In comparison to the leaf surfaces, the most abundant compounds in the whole leaf tissues (W and W-S) were the phenolic acids FeA and CA and the flavonoid DS, followed by lower levels of D, RA, LS and AS. This profile is similar to that of the *Z*. *noltii* chemotypes from the Atlantic Ocean and Mediterranean Sea, distinguished by a high ratio of DS versus RA and AS^37^.

DESI-IMS provided critical information on the spatial distribution of eelgrass surface metabolites. DESI-IMS analyses require very flat surfaces^27^, a feature not inherent to marine macrophytes. This problem was overcome by using leaf imprints that allowed efficient imaging and confirmed the presence of a complex chemistry on the eelgrass leaf surface. In agreement with the LC-MS results, DS was the most abundant surface-associated phenolic compound detected by DESI-IMS, followed by lower levels of the other flavonoids and the phenolic acids ZA and RA (Fig. 2B–F). The unique distribution of all phenolics along the midvein may suggest their internal transport by phloem to different tissues (e.g. rhizomes), whereas higher accumulation at the apex may be linked to a wide range of ecological functions, e.g. deterrence against pathogens or herbivores^16,18,19,39^. In addition, DESI-IMS revealed the presence of carboxylic acids and FAs possibly derivatives of membrane phospholipids, as those found by MN (Supplementary Table S2). The surface distribution pattern of these FAs suggests a structural role. The most abundant compound, palmitic acid, was evenly distributed over the surface, except for the midvein area. Other FAs and carboxylic acids accumulated around the margins of the leaf-blade. For instance, myristic acid formed a layer at the lower lamina periphery and around the apex (Fig. 3B) in the proximity of necrotic cells observed by light-microscopy (Supplementary Figs S2 and S19). Together, palmitic and myristic acids appear to constitute a surface lipid ‘*tunic*’ contributing, along with osmolytes e.g. trehalose^5,40^, to the stabilization and osmotic adjustment of membrane bilayer structures, thus protecting eelgrass epidermis against abiotic stresses, such as heat and saline environment^41,42^.

The high chemical diversity, differential spatial distribution and bioactivity profile of these surface metabolites indicate diverse ecological functions in eelgrass. In higher plants, overlapping functions between ‘primary’ and secondary’ metabolites are broadly demonstrated, including the role of phenolics in pollination, allelopathy, or symbiosis, and that of FAs in plant defence and immunity^21,43–45^. In a previous study, we showed the inhibitory activity of sulfated flavonoids from *Z*. *marina* leaf surface extracts against bacterial settlement^19^. In this study, both flavonoids and phenolic acids in their desulfated forms appeared to play complementary roles in the eelgrass protection against fouling yeasts. Leaf surface concentrations of DS, in its desulfated form (D) were found to inhibit settlement of microfoulers. Similar patterns observed for AS and LS indicated that these surface-associated flavonoids also play a role against fouling at natural surface concentrations (3–6 nmol.cm^−2^) comparable to those of surface-associated bromophycolides in the red seaweed *Callophycus serratus*^29^. Interestingly, the antioxidant and cytotoxic capacity of desulfated flavonoids is enhanced compared to their sulfated counterparts^20,46,47^. Thus, sulfate conjugation may not be directly involved in the bioactivity of these secondary metabolites but rather play a physiological function, for instance to facilitate metabolite cellular and/or long-distance transport within the plant^48,49^. Similarly, a protective role for ZA was suggested in our study by the activity of its desulfated form, *p-*Co, which is released *in vivo* enzymatically and was shown to be responsible for the antifouling properties of the compound^34^.

Although RA exhibited no bioactivity here, we previously observed its antisettlement activity toward bacterial microfoulers^19^. Several other functions, e.g. antimicrobial, nematicidal and antioxidant, have been attributed to RA^18,50,51^. In addition to the phenolic compounds distributed on the leaf surface, bioactive phenylpropanoic acids, such as FeA and CA, were highly abundant in the whole leaf extracts, suggesting their role in controlling the spread of microbial infection within the eelgrass inner tissues^16,18,52–54^.

Eelgrass phenolics, including those annotated in the MN, are quite unlikely to be synthesized by marine epibionts or symbiotic microorganisms, as they are common in seagrasses^18–21^ and land plants^52,55^. In addition to the phenolic acids and flavonoids commonly found in eelgrass and other seagrasses^18^, putative annotations by MN in our study suggested the presence of other phenolic compounds, such as isoflavone glycosides. Isoflavonoids have never been described from *Zostera* sp. so far, but the enzyme isoflavone reductase specific for isoflavonoid biosynthesis was shown to be over-expressed during heat-stress response in the seagrass *Z*. *noltii*^56^. Compared to secondary metabolites, it is difficult to establish the actual origin of FAs as they are ubiquitous components of all plant and bacterial cell membranes. Similarly to what is observed in seaweeds^24,57^, lipids from eelgrass membranes could be released on the surface by hydrolysis, as supported by the MN annotation (e.g. of FA esters and lyso-phosphatidylcholine). Comparison of the DESI-IMS surface intensities indicated that primary FAs (e.g. palmitic and myristic acids) on the eelgrass surfaces were even more abundant than the phenolics. In addition to a structural function, these FAs in eelgrass appear to play a defensive role against microfoulers, as suggested by our bioassays. Both saturated and unsaturated FAs have been previously reported as antimicrobial and antifouling agents^58,59^ and could collectively protect the eelgrass surfaces against water-borne pathogens. As other higher plants, eelgrass may further exploit lipid metabolism to activate systemically acquired resistance and induce chemical defence against pathogens. For instance, azelaic acid is involved in priming of defence responses via the lipid transfer protein AZELAIC ACID INDUCED 1 (AZI1)^45^. Blasting of the *AZI1 Arabidopsis* sequence produced a significant alignment with the locus ZA008351 (Supplementary Table S10), encoding a lipid transfer protein with a possibly similar function in *Z*. *marina*.

**Figure 4:**
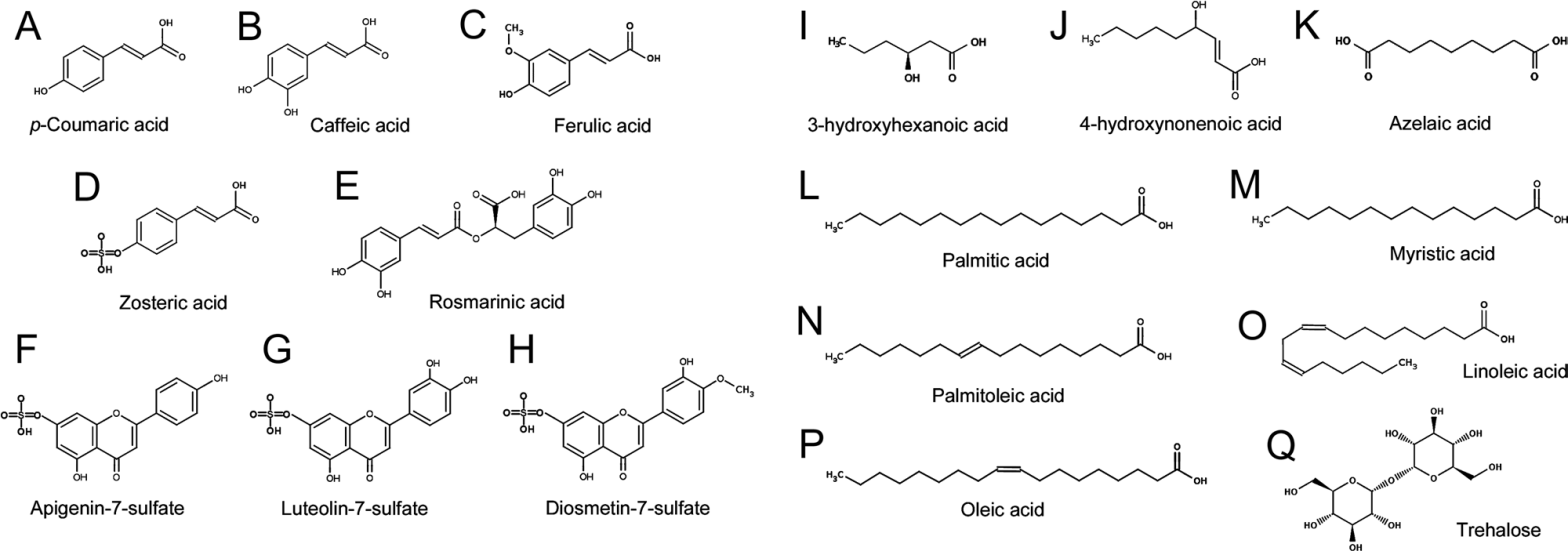
The major metabolites of *Z*. *marina* identified by LC-MS/MS and/or DESI-IMS. (**A–H**) Phenolic compounds, including the sulfated flavonoids (**F-H**). (**I**–**K**) Aliphatic carboxylic acids, including azelaic acid (**K**). (**L**–**P**) Fatty acids. (**Q**) Trehalose.

**Figure 5:**
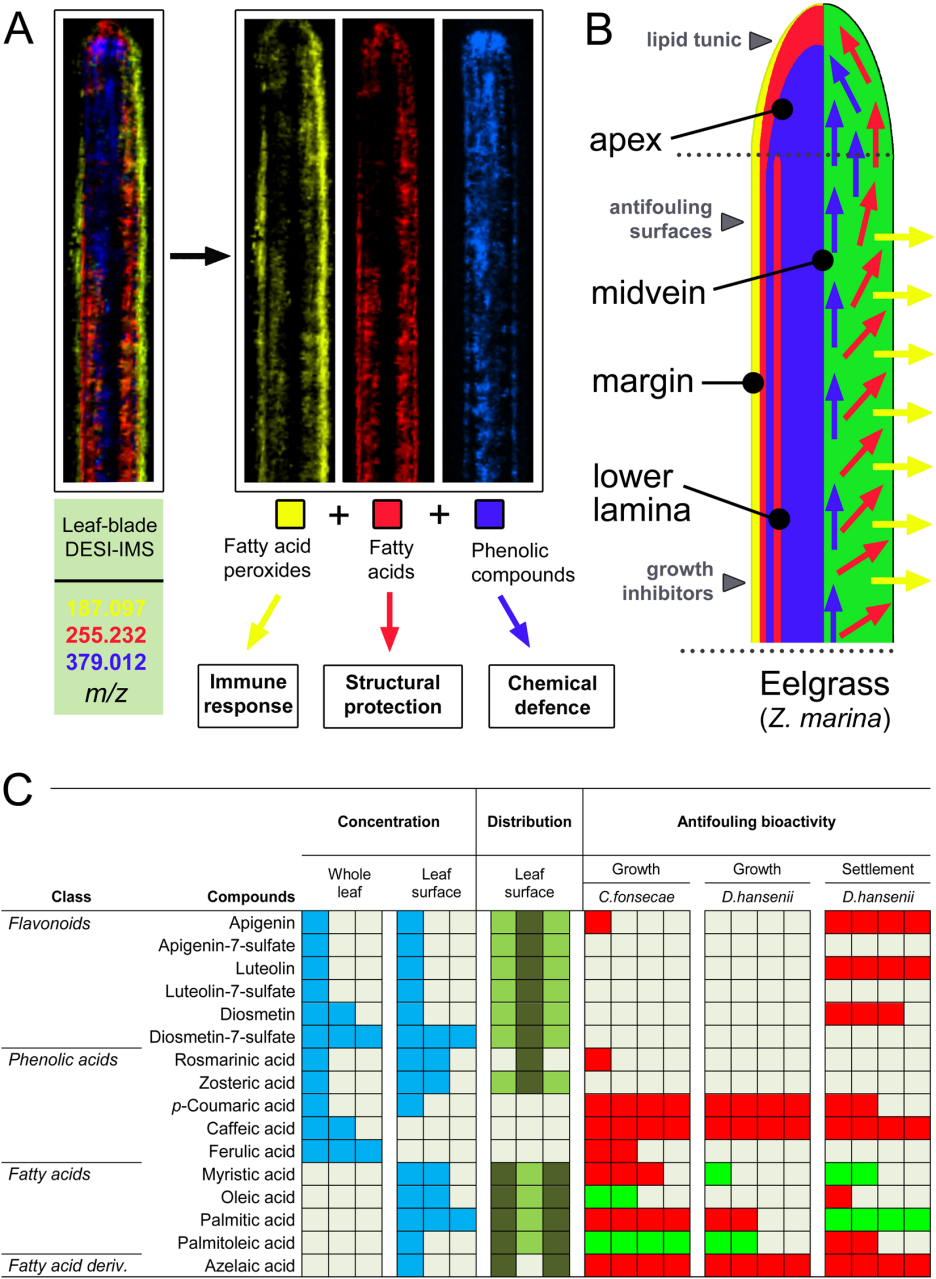
Eelgrass chemical defence against microbial foulers. (**A**) DESI-IMS scanned leaf-blade showing superimposition of azelaic acid, palmitic acid, and DS, representing three major classes of leaf surface-associated metabolites: FA peroxides (yellow), FAs (red), and phenolic compounds (blue). (**B**) Model suggesting differential functions of *Z*. *marina* surface metabolites. Surface metabolites may collectively confer physical and chemical defence, providing antifouling activity, antimicrobial activity and a protective lipid ‘*tunic*’ layer. (**C**) Visual summary of the defence metabolites found in this study for *Z*. *marina*, showing concentrations of flavonoids, phenolic acids, and fatty acids in the whole leaf and on the leaf surface, reported as relative abundances (in blue, from low to high abundance as one to three squares). The DESI-IMS leaf surface distribution for each metabolite is reported as relative abundances (light to dark green) on the midvein (central square) or margin (outer squares). The antifouling bioactivity for each of the metabolites tested against growth and settlement of microbial foulers is reported relative to the percentage of inhibition (red) or activation (green) as measured in the bioassay (see Supplementary Tables S7–S9).

Combination of untargeted metabolomics with chemical imaging and bioassays may represent a powerful strategy to address ecological questions (e.g. surface-based chemical signalling mechanisms and the nature of signalling molecules) and to uncover complex chemical interactions (e.g. host-microbe, host-pathogen and microbe-microbe) taking place on biological surfaces. Overall, this study shows that eelgrass *Z*. *marina* has a complex surface-associated chemistry comprised of both primary and secondary metabolites (Fig. 4). Our results suggest different functional roles and variable ecological targets of the phenolics and FAs on the eelgrass surface, indicating a higher defence capability against epibiosis and fouling in specific surface regions (Fig. 5). The combined chemical and bioactivity information shown herein suggests that phenolics and possibly other compounds (e.g. FAs and trehalose)^5^ are synthesized intracellularly by *Z*. *marina* and then transported and secreted to the surface. Although the exact release mechanisms are still unknown, compounds could be exported to the surface via exocytosis or protein channels^60^, or alternatively at the ingrowth interface of transfer cells between the inner vascular parenchyma and the outer leaf surface^36,61,62^. We propose a model highlighting the potential functions of the surface-associated metabolites in eelgrass (Fig. 5). This metabolic system may allow for a targeted control of both biotic defence (against pathogens and predators) and abiotic protection (against high salinity and temperatures). Thus, the evolutionary loss of the cuticle in seagrasses as a major defensive structure against microorganisms may be compensated with a more complex metabolic mechanism that allows finely tuned surface interactions in sections of the same leaf that are of the same age, but differently exposed to hydrodynamics (e.g. leaf margin *versus* leaf center). Future studies will aim at understanding the additional roles of these surface metabolites in chemical signalling, chemical adaptation, and in-depth ecological interaction strategies of *Z*. *marina*.

## Materials and Methods

### Sampling of the eelgrass material

Eelgrass *Z*. *marina* (Fig. 1A) was collected in September 2017 from the Baltic Sea coast of the Kiel Fjord, Falckenstein Beach, Germany (54°23′38.1″N, 10°11′23.4″E) (Supplementary Fig. S1). Plants were located at −1 m depth from the water surface. At the time of collection (10:00–12:00 a.m.) the recorded temperature was 14 °C, the salinity 1.7% (17 PSU), and the pH 7.7. Collected plants were immediately placed in plastic bags and transported inside a thermal box to laboratory. Plants were rinsed by quick dipping into artificial seawater adjusted to the salinity of the Baltic Sea (1.7% Instant Ocean® in milliQ water).

### Surface and whole leaf extractions

The eelgrass leaf surface was extracted by solid-phase adsorption (C18) as described by Cirri *et al*.^24^, or by solvent dipping (S) as described by Guan *et al*.^19^. Briefly, for the solid-phase extract, intact leaves from 30 plants were covered evenly with 20 g of C18 material (Sepra-E powder, particle size 50 μm, pore size 65 Å, Phenomenex®, California, USA) and agitated inside a round-bottom flask. The C18 material was rinsed from the leaves with milliQ water (200 mL) into a glass funnel with a filter and eluted twice with MeOH (200 mL) under vacuum. The MeOH phase was evaporated to dryness using Syncore® Polyvap R-12 (BÜCHI Labortechnik AG, Switzerland). For the solvent dipping extracts, isopropanol (IPA) dipping for 5 sec was found to be optimal, as it did not cause any chlorophyll saturation or leaf surface damage (Supplementary Fig. S3). Ten surface extract replicates (S) were prepared from individual plants by dipping four vegetative leaves (corresponding to a total leaf surface of 236.8 cm^2^) into IPA (100 mL) in a measuring cylinder. For the whole leaf (W) and the surface-free (W-S) extracts, freeze-dried and manually ground material obtained from four vegetative leaves (0.3 g.DW) was extracted by MeOH (5 min) using the automated Accelerated Solvent Extraction (ASE) Dionex® 350 (Thermo Fisher Scientific). Parameters of the automated system were set as follows: temperature 40 °C, rinse volume 30%, purge time 100 sec, water pre-rinse 10 min (3 cycles) performing extraction steps with MeOH for 5 min (1 cycle). Ten whole leaf extract replicates (W) were prepared from individual plants using this ASE method. The surface-free extracts (W-S) were prepared by applying the same method using the air-dried residual eelgrass material after IPA dipping (10 replicates). All extracts were evaporated to dryness using Syncore® Polyvap R-12 (BÜCHI Labortechnik AG, Switzerland).

### Bioassays against microbial foulers

Dose-response experiments for yeast growth or settlement were conducted as previously described^19^. 96-well plates were impregnated with eelgrass extracts or pure compounds pipetting into the wells stock solutions dissolved in either methanol (extracts) or ethanol (pure compounds). Stock solutions were added to a final volume of 100 µl and solvents evaporated in vacuum, resulting in the impregnation of a 0.955 cm^2^ surface per well. Concentrations of leaf surface extracts were related to naturally occurring concentrations on *Z*. *marina* surfaces assuming a leaf area/fresh-weight ratio of 78.99 cm^2^.g^−1^, as described by Guan *et al*.^19^. Correspondingly, a 1-fold natural concentration of surface extract was tested when extract obtained from 0.955 cm^2^ leaf surface was impregnated onto 0.955 cm² well surface. Concentrations of tissue extracts were dosed based on the assumption of a dry-weight/fresh-weight proportion of 10%. Correspondingly, a 1-fold natural concentration of tissue extract was tested when extract obtained from 10 mg dry weight was present in the final volume of 100 µl. The yeasts were maintained at 25 °C on a shaker in liquid medium containing: 3 g of yeast extract, 3 g of malt extract, 5 g of peptone, 10 g of glucose, and sea salt 3%. For growth bioassays, 100 µl medium containing yeast cells at 0.07–0.17 initial OD_610_ were pipetted into the wells. Plates were incubated at 25 °C on a shaker in darkness and OD_610_ was repeatedly measured over 20 h. Exponential growth curves were fitted with the GraphPad Prism 5.0 software package to OD data time series obtained for each well allowing determination of division rates. Division rates obtained for single wells with addition of compounds were then related to mean division rates obtained for six control wells without such addition. Settlement assays were conducted as described by Guan *et al*.^19^. Briefly, aliquots of yeast liquid cultures were pipetted into the wells. After 2 h, the wells were emptied and cells attached to the walls were stained with Calcofluor white for 10 min. The unattached cells and the excess dye were removed by rinsing with sterile seawater, and the fluorescence of stained cells attached to the wells was measured at 350 nm excitation and 430 nm emission. Dose-response curves were generated with GraphPad Prism 5.0 using growth or settlement data obtained for different concentrations and fitting a logistic function, which allowed for the determination of the EC_50_ and its 95% confidence interval. Additional information on the methods for isolation of the yeast strains is provided in the supplementary material.

### LC-MS/MS-based metabolomics studies

Eelgrass extracts were diluted with MeOH:water (1:1) mixture to a final concentration of 1 mg/mL and 1 μL was injected to a Acquity UPLC coupled to a Xevo G2-XS QTOF-MS (Waters®, Massachusetts, USA) operated with MassLynx® software (v4.1). Chromatography was achieved on a C18 column (Acquity UPLC HSS T3, 1.8 μm, 2.1 × 100 mm, Waters®) operating at 40 °C. Eluents were water (A1) and acetonitrile (B1) with 0.1% formic acid (v/v), both UHLPC grade (VWR®, Pennsylvania, USA), and were infused as binary mobile phase at a flow rate of 0.5 mL/min with a gradient of 99:1 (initial condition), 99:1 (1 min), 1:99 (9 min), and 0:100 (12 min) followed by column wash and reconditioning (3 min). The total chromatography time for each sample was 15 min. The MS and MS/MS spectra of the eluting compounds were detected in negative ionization mode for the ion mass range *m/z* [M-H]^−^ 50–1200. The QTOF-MS source temperature was set to 150 °C, capillary voltage 1 kV, sampling cone voltage 40 V, cone gas flow 50 L.h^−1^, desolvation temperature 600 °C, and desolvation gas flow 1200 L/h. MS/MS spectra were generated in data dependent analysis (DDA) mode using collision energies ramps of 6–9 eV for low mass (50 Da) and 60–80 eV for high mass (1200 Da), respectively. Data were acquired in sensitivity mode scanning every 0.1 sec (0.01 sec interscan delay) with data format centroid. For accurate mass measurements we used the reference lock-spray mass as the negative ion of Leucine enkephalin (*m/z* [M-H]^−^ 554.261). Phenolic compounds were quantified by comparing the peak area of the extract samples with those of the pure standards, *p*-coumaric acid, rosmarinic acid, apigenin, luteolin, and diosmetin, measured at different concentrations (from 1 ng.mL^−1^ to 25 µg.mL^−1^). Four technical replicates were injected in quadruplicate (1 µL) into the UHPLC-QTOF-MS system using a linear gradient: 99% A1 (0–7 min), 0% A1 (7–8 min) followed by column reconditioning to 11 min. The same flow rate (0.5 mL.min^−1^) and MS conditions were used for analysis of the extracts.

A calibration curve was calculated based on the *m/z* peak intensity for each compound (luteolin: *y* = 31313*x* and R² = 0.992; rosmarinic acid: *y* = 2e^6^*x* and R² = 0.985; diosmetin: *y* = 1e^7^*x* and R² = 0.998; apigenin: *y* = 15593*x* and R² = 0.985; *p*-coumaric acid: *y* = 2e^6^*x* and R² = 0.999) (see also Supplementary Fig. S15). The concentration of each phenolic compound was measured in the eelgrass extracts (i.e. µg or ng.mL^−1^) (Fig. 1D and E) and related to moles per dry weight for the whole leaf extracts (µmol.gDW^−1^), and to moles per leaf surface for the surface extracts (nmol.cm^−2^) (Supplementary Table S1). Additional information on the methods for data processing, metabolite annotation, and visualization is provided in the supplementary material.

### Chemical imaging (DESI-IMS)

Leaf imprints obtained by pressing eelgrass surfaces directly on clean glass slides (dim. 25 × 75 × 1 mm, SuperFrost Ultra Plus®, Thermo Fisher Scientific) were analyzed with a DESI source (Prosolia®, Indianapolis, USA) connected to the QTOF-MS. The glass slides were placed on the DESI-IMS 2D moving stage controlled using the software Omni Spray 2D v2.0.1 (Prosolia®). Electrospray was achieved at 5 kV voltage with 0.5 MPa of nitrogen gas and a solvent mixture of MeOH and water (95:5 v/v) infused through a glass syringe (1 mL, Ø4.61 mm) using an external hydraulic pump at constant flow rate 1.5 μL min^−1^. The emitter capillary was a pre-cut, non-coated, fused-silica PicoTip/TaperTip® (40 mm, Ø 360 µm OD × 20 µm ID; New Objective®, Massachusetts, USA). The geometry of the system was set to: sprayer angle 75°, emitter protrusion 1 mm, distance sprayer to surface 1.5 mm, surface to MS-inlet 0.1 mm, and emitter to MS-inlet 6 mm. Surfaces were rastered at a scan rate of 150 μm.sec^−1^ and 150 μm lateral resolution (pixel size). Data were acquired in sensitivity mode (*m/z* 100–1500, negative ionization) with data format continuum, and were analysed using the software HDImaging v1.3.5 (Waters®) and OpenMSI^33^ (https://openmsi.nersc.gov/). Additional information on processing and visualization methods is provided in the Supplementary Material.

### Statistical analyses

Multivariate analysis was performed with unsupervised or supervised models, e.g. principal component analysis (PCA) and partial least square (PLS), discriminant analysis (DA) using SIMCA® v14 (Umetrics®, Umeå, Sweden). These methods were used to determine whether a reliable separation between the samples could be achieved on the basis of their metabolite profile (explanatory X-variables) describing the difference in the response (dependent Y-variables) associated with extract conditions (C18, S, W, W-S) or, in case of the DESI-IMS imaging data, with specific ROI of the leaf-blades. The strengths of the models is estimated by the amount of cumulative (cum) variation in X computed in the projection that is predictive and correlated to Y, and is reported by the terms R^2^X (cum) and R^2^Y (cum) respectively, with a maximum value of 1.0. The X-variables were scaled using Pareto method, i.e. mean-centered and divided by the square root of the standard deviation. The models were fitted to the minimum number of principal components (PC), or in case of regression models the latent variables (LVs), corresponding to the highest value of predicted variation - Q^2^ (cum).

## Supplementary information

Supplementary information

## Data Availability

The MS/MS dataset used for the analysis was deposited in the MassIVE Public GNPS database (http://massive.ucsd.edu). under access number MSV000081679. Imaging data deposited on the NERSC platform OpenMSI (https://openmsi.nersc.gov/). are available for online high-performance visualization and analysis (https://goo.gl/523GEU). Datasets obtained in the bioassays can be downloaded from the depository PANGAEA (https://www.pangaea.de/) under the ID number 10.1594/PANGAEA.897495.
