# Supplementary material for: Surface chemical defence of the eelgrass Zostera marina against microbial foulers
Source: Sci Rep. 2019 Feb 26;9:3323. doi: 10.1038/s41598-019-39212-3 (PMC6389981; doi:10.1038/s41598-019-39212-3)
Supplement: Supplementary file 1 — Supplementary information [file 41598_2019_39212_MOESM1_ESM.pdf]

Supplementary information

Surface chemical defence of the eelgrass *Zostera marina* against microbial foulers

Stefano Papazian[1], Delphine Parrot[1], Barbora Burýšková[2], Florian Weinberger[2], Deniz Tasdemir[1,3,*]

[1] GEOMAR Centre for Marine Biotechnology, Research Unit Marine Natural Products Chemistry, GEOMAR Helmholtz Centre for Ocean Research Kiel, Am Kiel Kanal 44, 24106 Kiel, Germany
[2] Research Unit Marine Benthic Ecology, GEOMAR Helmholtz Centre for Ocean Research Kiel, Düsternbrooker Weg 20, 24105 Kiel, Germany
[3] Kiel University, Christian-Albrechts-Platz 4, 24118 Kiel, Germany

Corresponding author: Deniz Tasdemir
Email: dtasdemir@geomar.de
ORCID ID: https://orcid.org/0000-0002-7841-6271

This PDF file includes:

**Additional Material and Methods**	**p. 2**
**Supplementary Figures** (Figure S1 to S28)	**p. 3-32**
**Supplementary Tables** (Table S1 to S10)	**p. 33-44**
**References**	**p. 45**

Data Processing and Visualization

Feature extraction, deconvolution, and filtering of the LC-MS raw data were performed with XCMS online (https://xcmsonline.scripps.edu/) and the Optimus workflow (KNIME Analytics Platform, v3.2.1) based on OpenMS algorithms[1]. Peak alignment and integration were executed in Matlab®. Further inspection of chromatographic single MS peaks and tandem fragmentation was achieved in MZmine2 (https://mzmine.github.io/)[2] and OptimusViewer (https://github.com/MolecularCartography), respectively. For processing and visualization of the DESI-IMS imaging data including feature extraction for selected region-of-interest (ROI; 3x3 pixels), the HDImaging and Masslynx (Waters®, Massachusetts, USA) software were used. Imaging data were then converted to imzML format and uploaded for web-based processing, storage, and high-performance visualization on the dedicated platform OpenMSI (https://openmsi.nersc.gov/)[3] hosted at the National Energy Research Scientific Computing Center (NERSC). DESI-IMS spectra were inspected in OpenMSI comparing the abundances and distribution of compounds across the leaf surface (Figure S21). Pixel intensities for each compound over the surface were measured with ImageJ software (https://imagej.nih.gov/ij/) relating average and maxima abundances to quantifications previously obtained by solvent dipping surface extraction method (Table S1).

Metabolome Annotation

Annotation of compounds detected by UHPLC-QTOF-MS and DESI-IMS was achieved comparing chemical information from reference literature of metabolites described in *Zostera* sp. (Table 1, main article) and the METLIN mass spectra depository of the Scripps Research Institute (https://metlin.scripps.edu)[4,5] considering a mass tolerance of 10 ppm, and METLIN *in silico* or experimental MS/MS fragmentation. The MS/MS spectral data was used to generate additional untargeted metabolite identification in Global Natural Products Social molecular networking (GNPS) (https://gnps.ucsd.edu)[6]. Acquired DDA data were converted to mzML format with MS-Convert[7] and uploaded in GNPS. The tandem MS molecular network was created using the GNPS data analysis workflow with precursor ion mass tolerance set to 0.5 Da and product ion tolerance to 0.3 Da. The minimum cosine score for network edges was set to 0.7 with 4 peaks as minimum matched spectra. Consensus spectra with less than 2 spectra were not included in the analysis. After filtering nodes from the extraction solvent background, putative compound identifications were assigned to the nodes based on comparisons between fragmentation patterns in the similarity network and spectral information available in the GNPS spectral libraries (Table S2). For visualization of the constructed network we used Cytoscape 3.6.1 (www.cytoscape.org/)[8]. Additional annotations for unknown compounds were predicted *in-silico* with SIRIUS by computing MS/MS fragmentation trees[9] (Table S3).

Isolation of Yeast Strains

Yeast isolate ZM14DH1 was isolated from eelgrass collected at Falckenstein Beach, in June 2014. Leaf sections were introduced under sterile conditions into test tubes containing 4 mL of autoclaved seawater and homogenized on ice with a sterilized Ultra Turrax. The homogenate was diluted with sterile seawater by factors of 10 and aliquots of each dilution step inoculated on yeast nutrient medium with 12 g Kobe-agar, 10 g Glucose, 5 g Peptone, 3 g yeast extract and 3 g maltose in 1 L Baltic Seawater (pH 5.8). After one week of incubation in darkness at 15 °C, the colonies were isolated and maintained on the same agar as described above. Yeast isolate KF921 was obtained from a seawater sample collected in August 1995 at the island Helgoland (North Sea, Germany) during the Victor Hensen cruise 95-21 by Dr. Karsten Schaumann (Alfred-Wegener Institute Helmholtz Centre for Polar and Marine Research, Bremerhaven, Germany). The isolation medium contained 1.0 g glucose, 0.5 g peptone, 0.1 g yeast extract and 20 g agar dissolved in 1 L North Sea water. DNA barcoding of both isolates allowed identifying them as *Cryptococcus fonsecae* DeGarcia, Zalar, Brizzio, Gunde-Cimerman & van Broock (strain KF921) and *Debaryomyces hansenii* (strain ZM14DH1). Stock cultures of all strains were maintained until they were used for the experiments either stored in liquid nitrogen (KF921) or at -80 °C (ZM14DH1).

Supplementary Figures

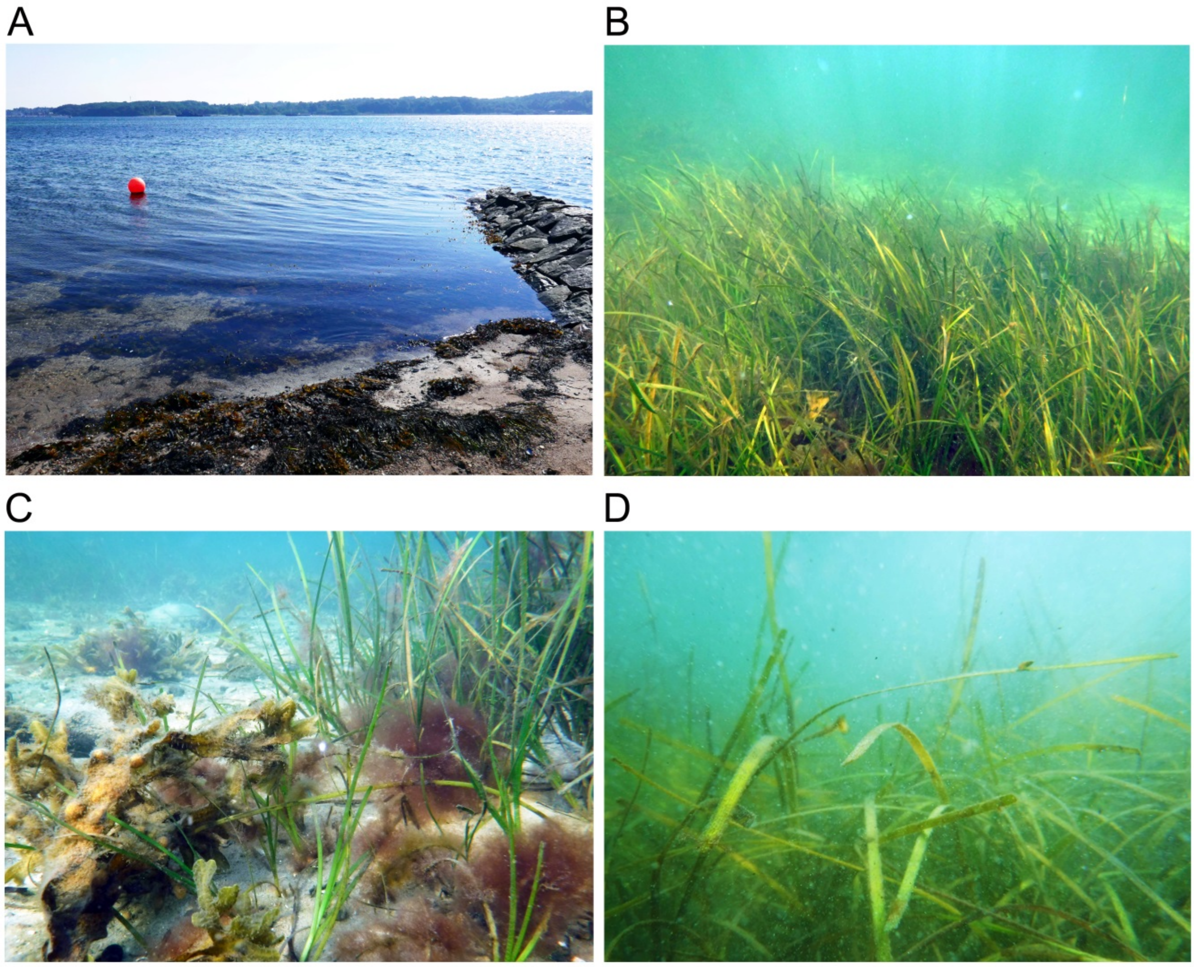

Figure S1. *Zostera marina* in the Baltic Sea. (***A***) Eelgrass meadow located at Falckenstein Beach, Kiel Fjord, Germany (54°23'38.1" N, 10°11'23.4"E). The specimens were collected (***B***) at a depth of -1 m, approximately at 50 m from the coast. At the time of collection (10:00-12:00 a.m.) in September 2017, the recorded water temperature was 14°C, the salinity was 1.7% (17 PSU), and the pH was 7.7. (***C***) Patches of eelgrass *Z. marina* were located in the proximity of other species important for the aquatic community of the Baltic Sea, such as the seaweeds *Gracilaria* sp. and *Fucus vesiculosus*. (***D***) Eelgrass plants displayed healthy and clean intact surfaces, with only minor presence of fouling and epiphytic growth. All photos by Stefano Papazian.

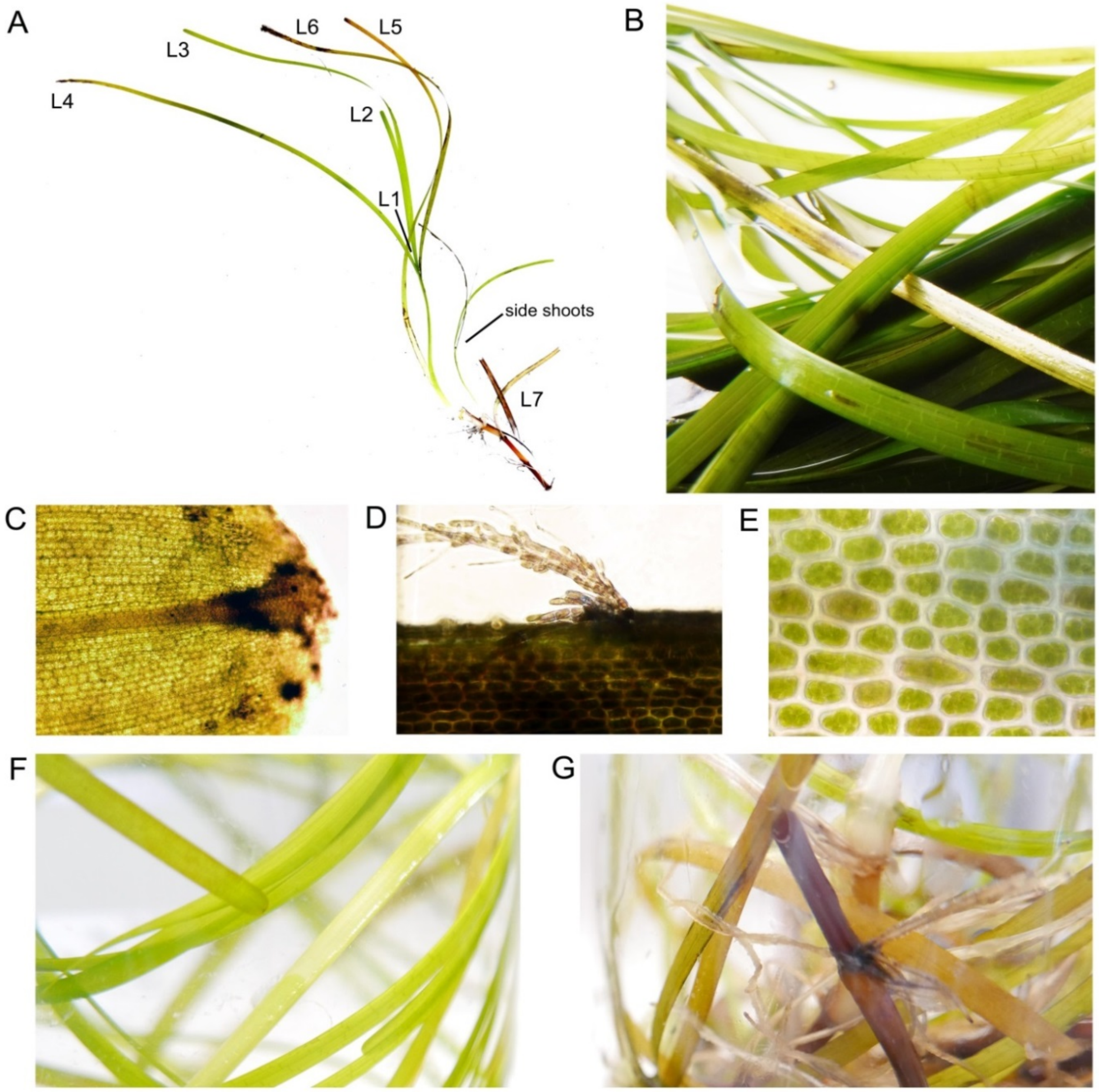

Figure S2. The physiology of *Zostera marina*. (***A***) Eelgrass plants usually comprised six or seven leaf-blades (L1-L7) radially developing from the core of the meristem towards the outside. (***B***) Inspection showed young vegetative leaves with healthy tissues and clean surfaces with no fouling. (***C-D***) Light microscopy confirmed intact and photosynthetically active leaf-blade surfaces. (***C***) In many specimens, a small necrotic region of central cells converging along the mid-vein towards the apex was typically visible at 10x magnification (***D***) Minor epiphytes were occasionally found growing on the edges of the leaf-blade visible at 40x magnification. (***E***) Inside single cells, chloroplasts were distinguishable at 100-200x magnification. (***F***) We selected the healthiest fully developed vegetative leaves from the inner layers, normally located between leaf positions one and four (L1-L4). (***G***) Senescing leaves. All photos by Stefano Papazian.

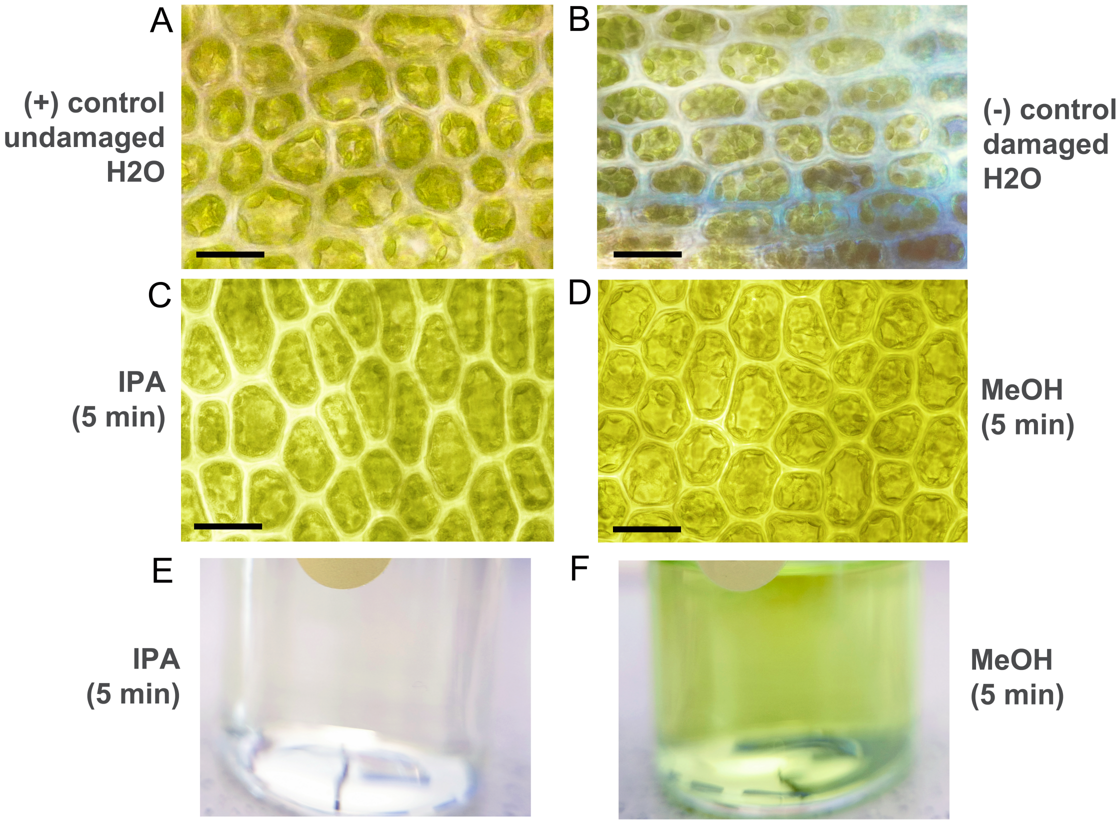

Figure S3. Eelgrass surfaces after extraction with isopropanol (IPA) or methanol (MeOH) as solvents. The effect of the solvents on the leaf surface integrity was assessed by staining for 30 min with Evans blue dye 0.05% solution in artificial seawater, following inspection with light microscopy[10]. (***A***) Positive control with undamaged eelgrass dipped into artificial seawater. (***B***) Negative control dipped into artificial seawater after mechanical damage of the eelgrass surface showing cell staining (blue). (***C-D***) Surface dipping with IPA or MeOH for 5 min, followed by staining and microscopy. Eelgrass surfaces after extraction appeared intact (no staining) but much stronger chloroplast bleaching was observed with MeOH compared to IPA, resulting in higher chlorophyll saturation in the solvent extract (***E-F***). All photos by Stefano Papazian.

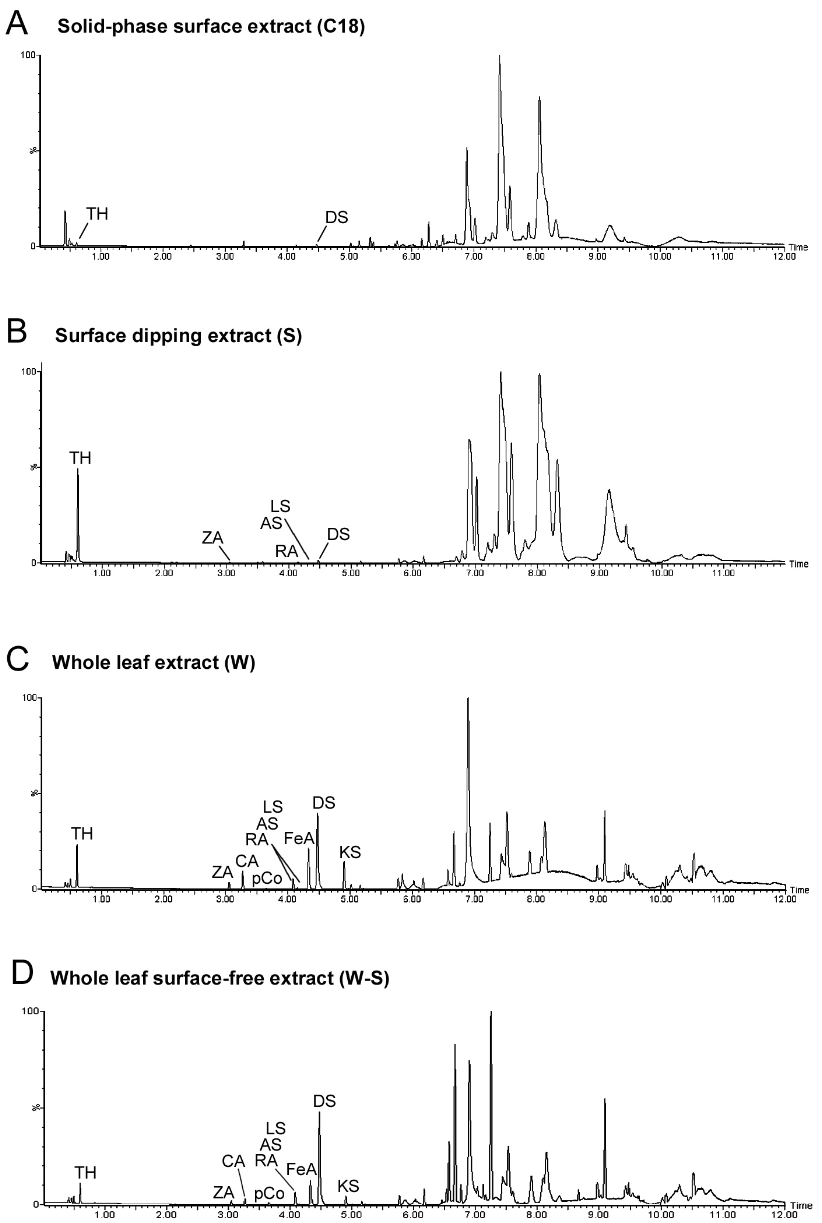

Figure S4. Comparative metabolome analysis of the eelgrass extracts. Base peak chromatograms show the comparative UHPLC-MS profiles of (***A***) surface solid-phase (C18), (***B***) surface solvent dipping (S), (***C***) whole leaf (W), and (***D***) whole leaf surface-free i.e., whole leaf after surface dipping (W-S) extracts of eelgrass *Z. marina*. UHPLC-QTOF-MS analyses were performed using a binary mobile phase of water and ACN, both with 0.1% formic acid (v/v) eluted at a flow rate of 0.5 mL/min (12 min). MS and MS/MS spectra of the eluting compounds were detected in negative ionization mode for the ion mass range *m/z* $[M-H]^-$ 50-1200. During reversed-phase separation with water and ACN gradient over a total run time of 12 min, several compounds eluted in the first part of the chromatogram (i.e. mid-polar region between 3-5 min), including the phenolic compounds zosteric acid (ZA), caffeic acid (CA), *p*-coumaric acid (pCo), ferulic acid (FA), rosmarinic acid (RA), and the sulfated flavonoids, apigenin-7-sulfate (AS), luteolin-7-sulfate (LS), diosmetin-7-sulfate (DS), and the putative kaempferol-7,4-dimethylether-3-O-sulfate (KS). At the very start of the elution (0.6 min), the disaccharide trehalose (TH) was also detected in the adduct form with formic acid. See the retention times, *m/z* ions, and metabolite IDs in the Table 1 (main article), quantifications in Table S1, and molecular networking annotations for fatty acids (6-11 min) in Table S2, and additional putative unknown *in silico* prediction in Table S3.

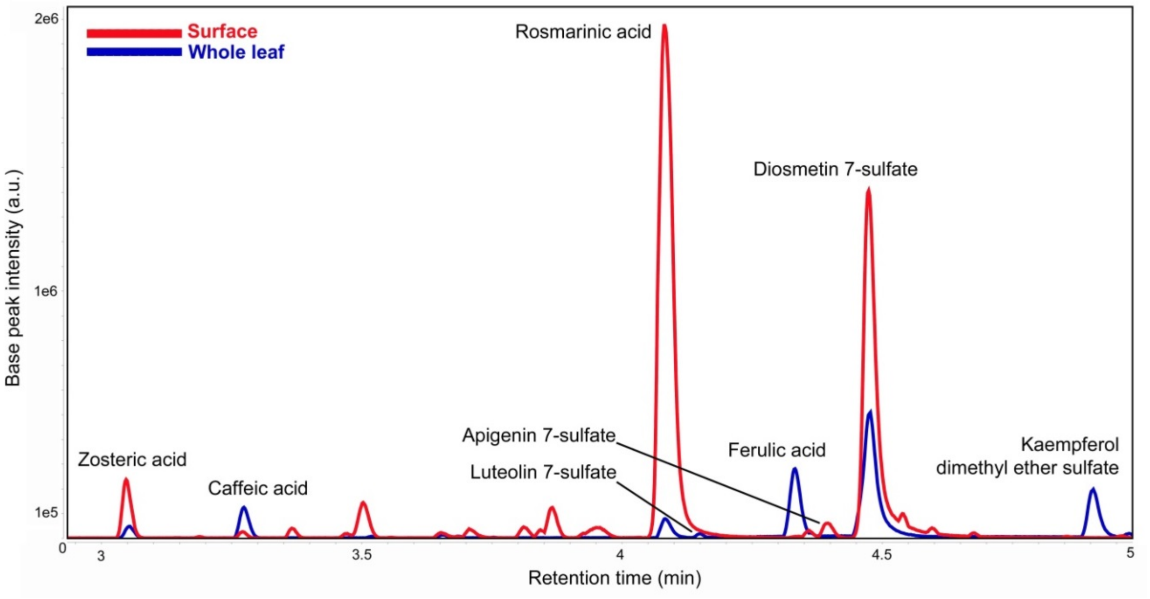

Figure S5. Phenolic compounds in the *Z. marina* surface dipping (S) and whole leaf (W) extracts. In total, ten biological replicates from individual plants were used to analyze the leaf surface and tissue extracts. The UHPLC-QTOF-MS chromatograms show the relative abundance of one S replicate with the highest phenolic compound concentrations (red), compared to a leaf W extract profile (blue). In all W replicates, the major phenolics (Table S1) were ferulic acid, caffeic acid and diosmetin-7-sulfate (DS). Similarly, DS was the most abundant phenolic compound in all S extracts, except for one S extract that contained very high levels of rosmarinic acid (RA), even exceeding the DS levels. High concentrations of RA was also detected in a second surface extract replicate albeit at lower levels than DS. All other S replicates displayed much lower levels of RA.

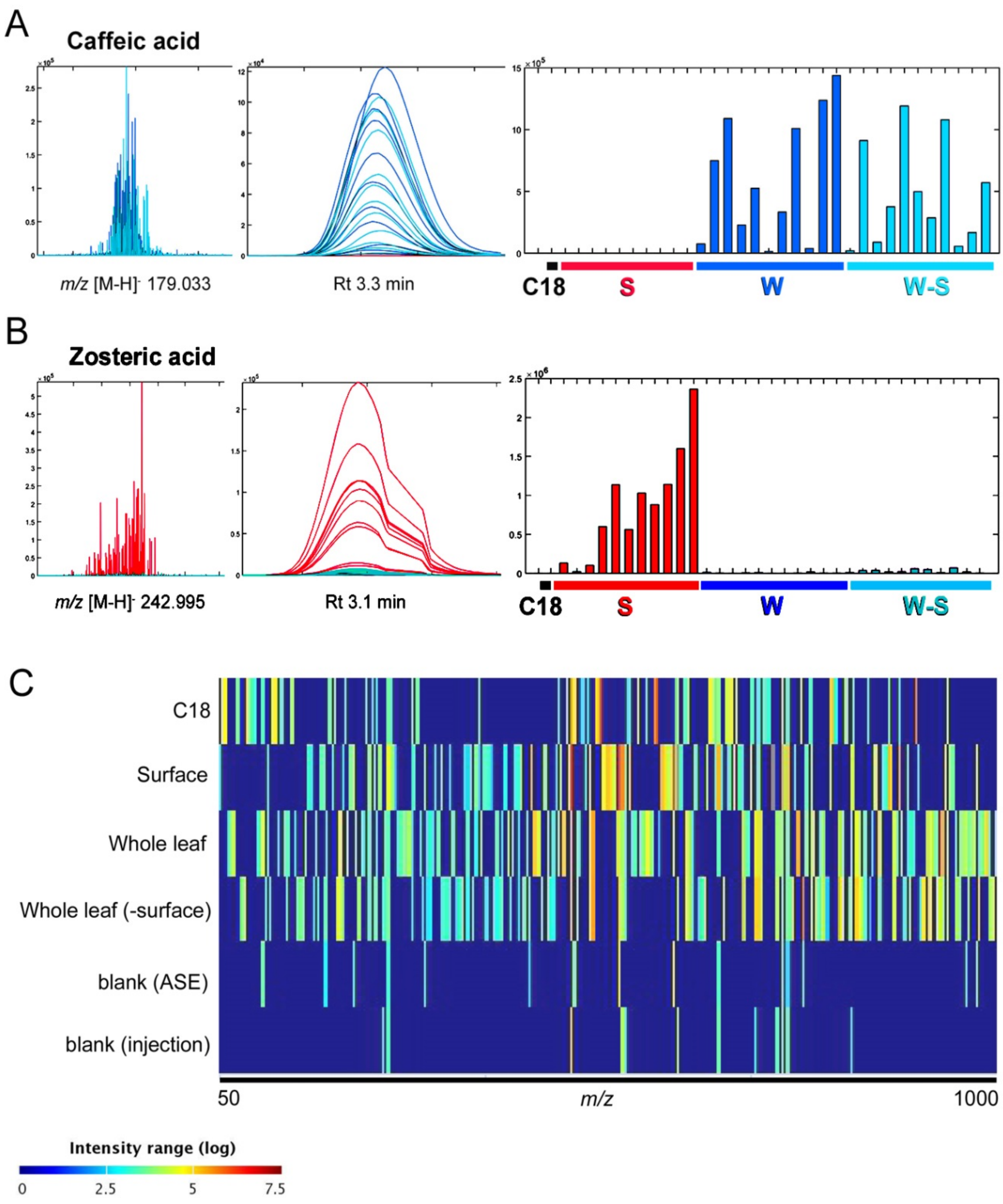

Figure S6. Qualitative and quantitative metabolite profiles of eelgrass extracts obtained by solid-phase surface adsorption (C18), surface dipping (S), whole leaf (W), and whole leaf after surface dipping (W-S). (***A-B***) Quantification of UHPLC-QTOF-MS chromatograms via peak area integration for each ion *m/z* feature performed in Matlab. Example reported for the phenolic metabolites (***A***) caffeic acid, and (***B***) zosteric acid, measured at higher abundances in whole leaf tissue (W, W-S) and surface (S) extracts, respectively. (***C***) Visualization in Optimus for MS and MS/MS feature distribution (*m/z* range 50-1000) comparing differences between all UHPLC-QTOF-MS extract profiles.

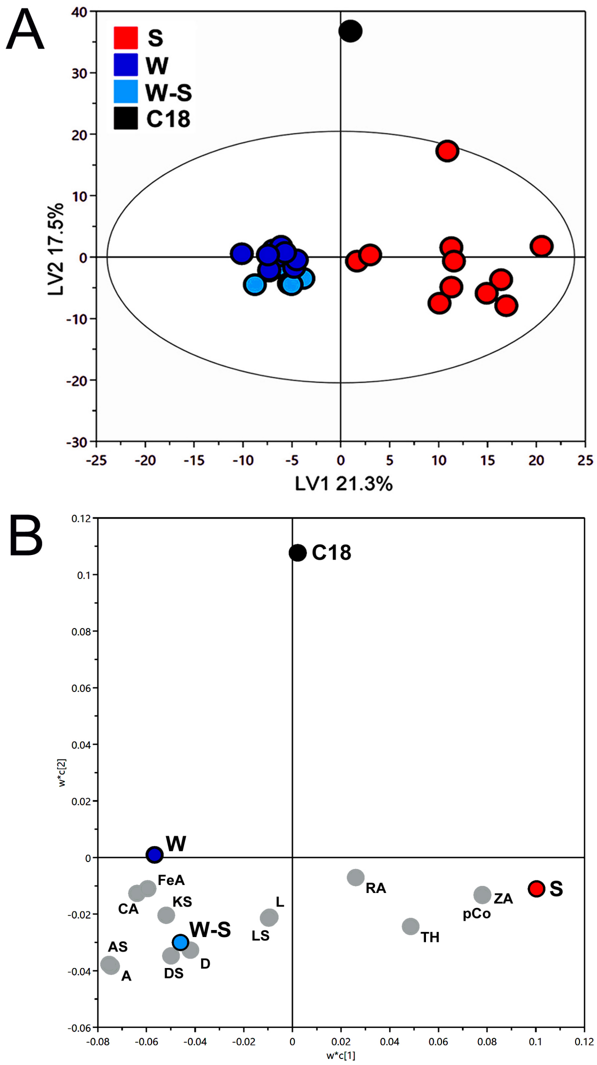

Figure S7. Supervised multivariate analysis (PLS-DA 3 components; see statistics in Table S5). (***A***) Scores, showing comparative extractions and LC-MS/MS metabolomics of surface solid-phase (C18), solvent dipping (S), whole leaf (W) and surface-free (W-S) extracts. (***B***) Loadings, showing the contribution to the model for the identified metabolites: *p*-coumaric acid (*p*-Co), apigenin (A), luteolin (L), apigenin-7-sulfate (AS), luteolin-7-sulfate (LS), caffeic acid (CA), ferulic acid (FeA), rosmarinic acid (RA), zosteric acid (ZA), diosmetin (D), diosmetin-7-sulfate (DS), kaempferol-7,4'-dimethylether-3-O-sulfate (KS, putative), and threalose (TH).

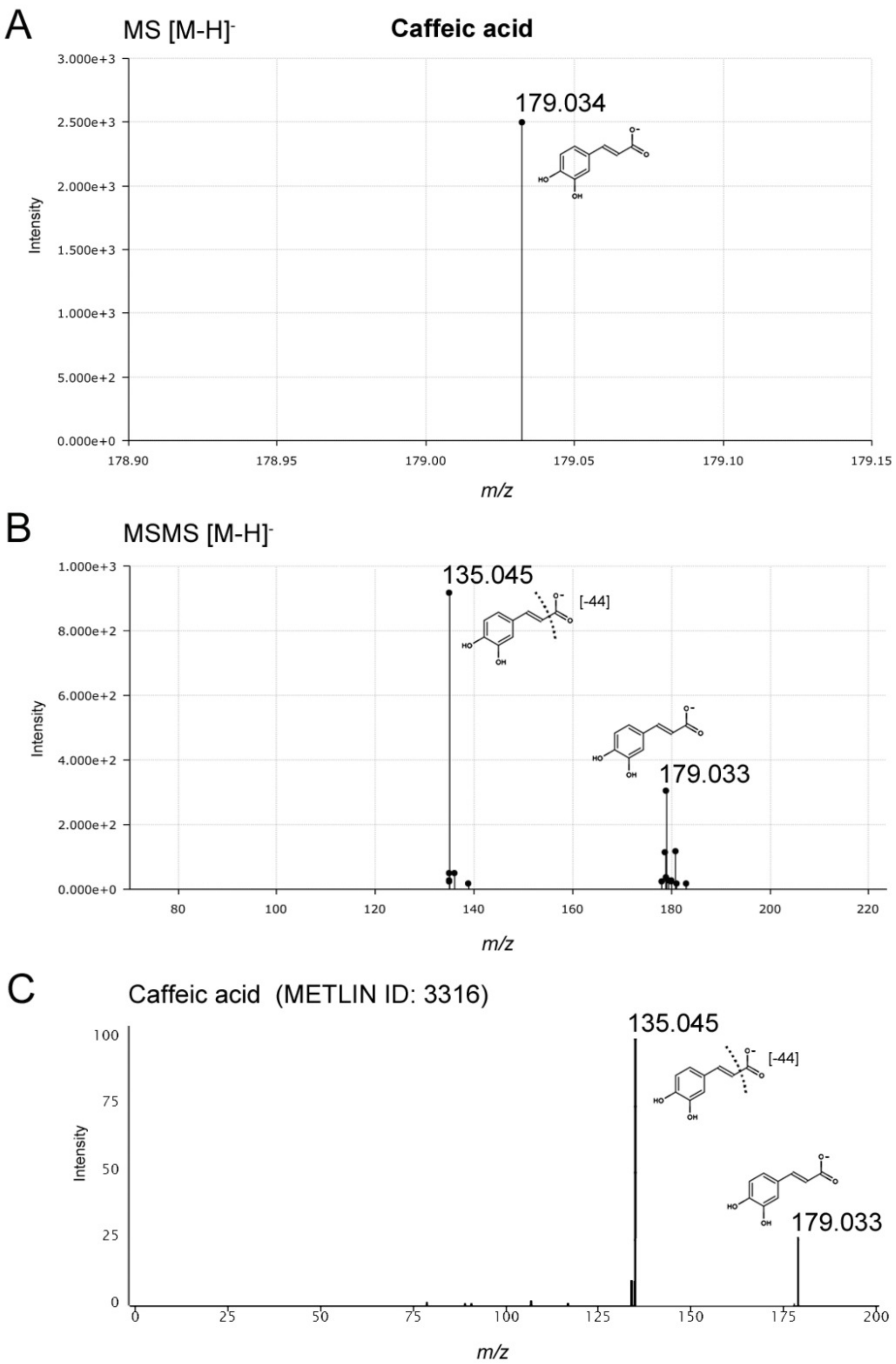

Figure S8. Targeted MS/MS identification of caffeic acid. (***A***) Caffeic acid (3,4-dihydroxy cinnamic acid) was detected by UHPLC-QTOF-MS in negative ionization mode as ion *m/z* $[M-H]^-$ 179.034 ($C_9H_7O_4$) at a retention time of 3.3 min. (***B***) MS/MS fragmentation (30-80eV) of the molecular ion with the loss of carboxylic acid moiety to form product ion *m/z* $[M-COO-H]^-$ 135.045 ($C_8H_7O_2$). (***C***) Caffeic acid experimental detection with UHPLC-QTOF-MS/MS as reported on the METLIN database (ID: 3316), showing molecular ion *m/z* $[M-H]^-$ 179.033 and fragment ion formation at *m/z* $[M]^-$ 135.045 (10-40 eV).

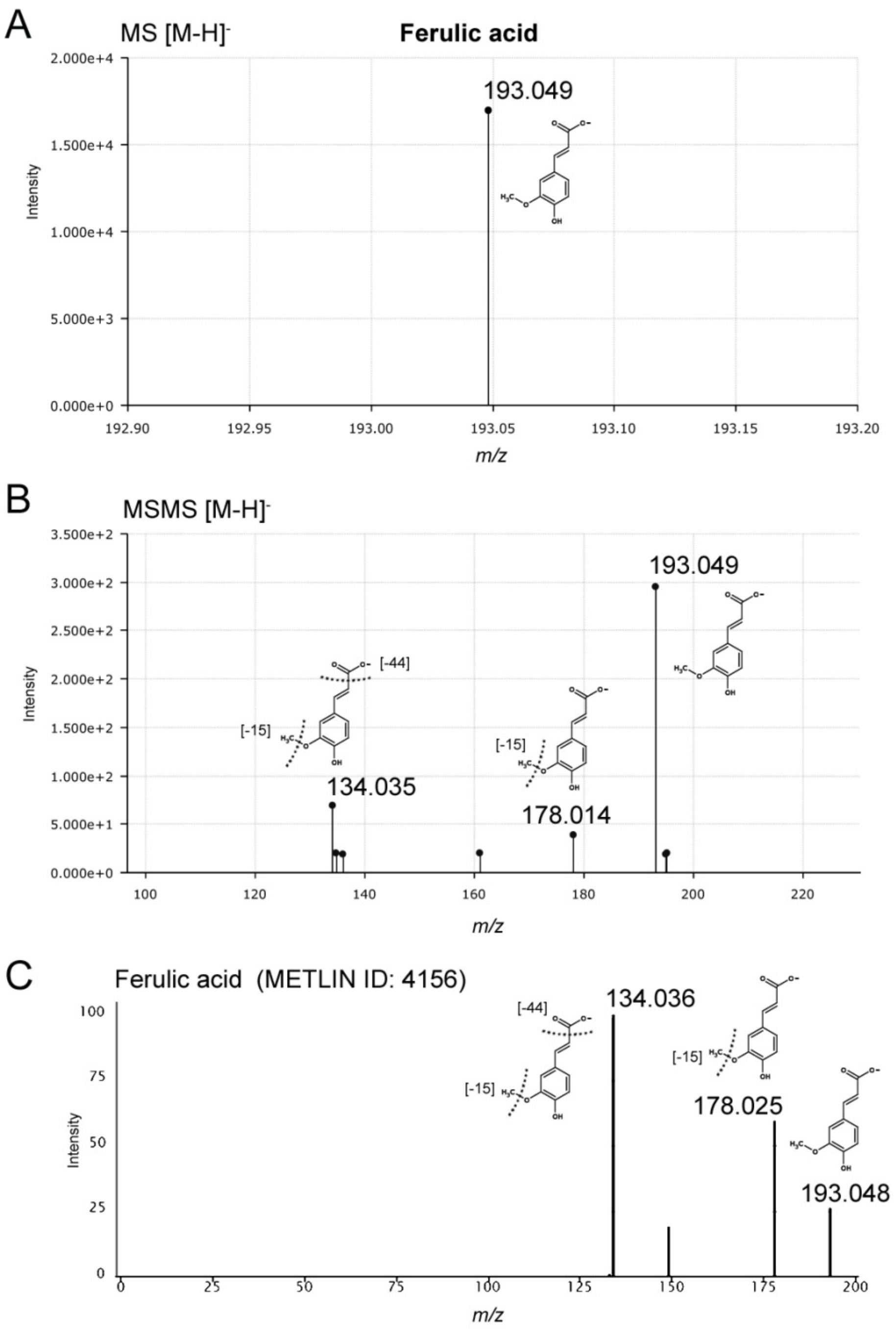

Figure S9. Targeted MS/MS identification of ferulic acid. (***A***) Ferulic acid was detected by UHPLC-QTOF-MS in negative ionization mode as ion *m/z* $[M\text{-}H]^-$ 193.049 ($C_{10}H_9O_4$) at a retention time of 4.3 min. (***B***) MS/MS fragmentation (30-80 eV) of the molecular ion with the loss of methyl group to form ion *m/z* $[M\text{-}H\text{-}CH_3]^-$ 178.014 ($C_9H_6O_4$) and further loss of the carboxylic acid moiety to form ion *m/z* $[M\text{-}H\text{-}CH_3\text{-}COO\text{-}]^-$ 134.035 ($C_8H_6O_2$). (***C***) Ferulic acid experimental UHPLC-QTOF-MS/MS detection as reported in the METLIN database (ID: 4156) showing of the molecular ion *m/z* $[M\text{-}H]^-$ 193.048 and formation of ion fragments (10-40 eV).

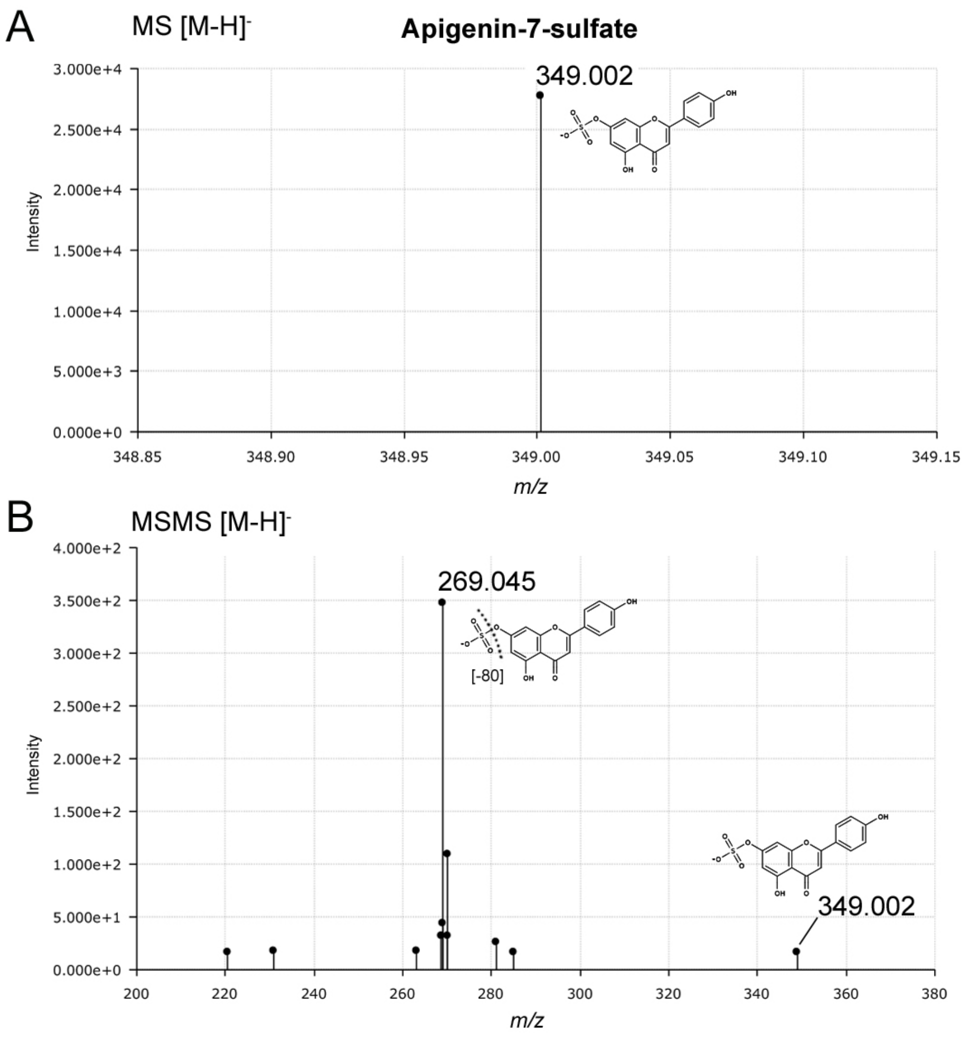

Figure S10. Targeted MS/MS identification of apigenin-7-sulfate. (***A***) Apigenin-7-sulfate was detected by UHPLC-QTOF-MS in negative ionization mode as *m/z* $[M\text{-}H]^-$ 349.002 ($C_{15}H_9O_8S$) at a retention time of 4.4 min. (***B***) MS/MS fragmentation (30-80eV) of the molecular ion with the loss of sulfate moiety to form the apigenin ion *m/z* $[M\text{-}SO_3\text{-}H]^-$ 269.045 ($C_{15}H_8O_6$). The same compound was reported in previous analyses on *Z. noltii* (see Table 1, main article). Apigenin-7-sulfate experimental UHPLC-QTOF-MS/MS detection reported in the METLIN database (ID: 48862) for the molecular ion *m/z* $[M\text{-}H]^-$ 349.002 and the predicted *in silico* fragmentation in positive mode yielding apigenin at *m/z* $[M+H]^+$ 271.060.

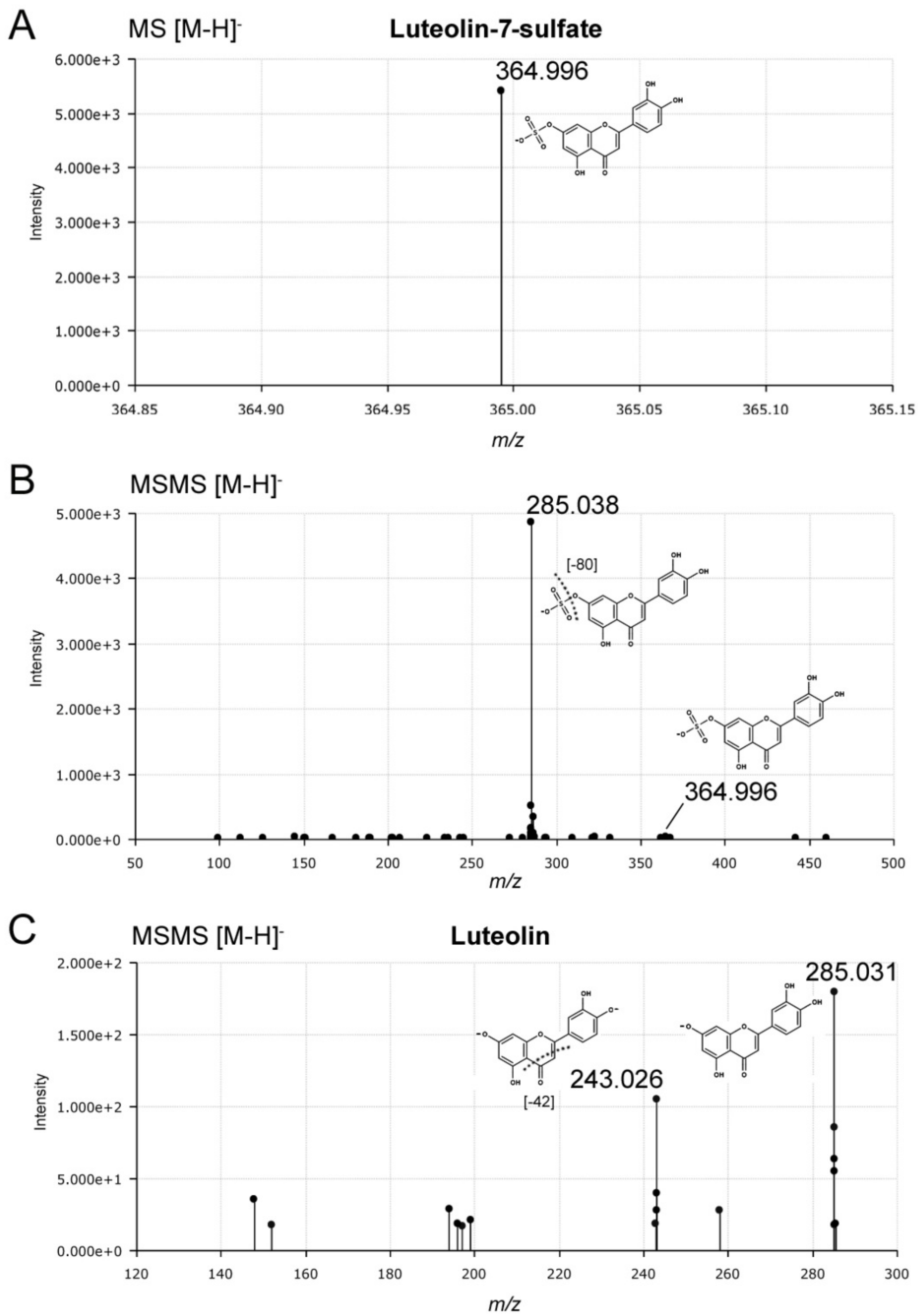

Figure S11. Targeted MS/MS identification of luteolin-7-sulfate. (***A***) Luteolin-7-sulfate was detected by UHPLC-QTOF-MS in negative ionization mode as *m/z* $[M\text{-}H]^-$ 364.996 ($C_{15}H_9O_9S$) at a retention time of 4.2 min. (***B***) MS/MS fragmentation (30-80eV) of the molecular ion with neutral loss of sulfate moiety to form luteolin ion *m/z* $[M\text{-}SO_3\text{-}H]^-$ 285.038 ($C_{15}H_9O_6$). The same fragmentation reported in previous analyses on *Z. marina* (see Table 1, main article). In comparison, luteolin-7-sulfate is reported in the METLIN database (ID: 49164) with UHPLC-QTOF-MS/MS experimental detection of the molecular ion *m/z* $[M\text{-}H]^-$ 364.996, and predicted *in silico* fragmentation spectra in positive mode producing luteolin ion *m/z* $[M\text{+}H]^+$ 287.055. (***C***) Luteolin molecular ion at *m/z* $[M\text{-}H]^-$ 285.031 ($C_{15}H_9O_6$) and detected MS/MS fragmentation with cleavage of the aromatic ring to form ion *m/z* $[M\text{-}H\text{-}C_2H_2O]^-$ 243.026 ($C_{13}H_7O_5$).

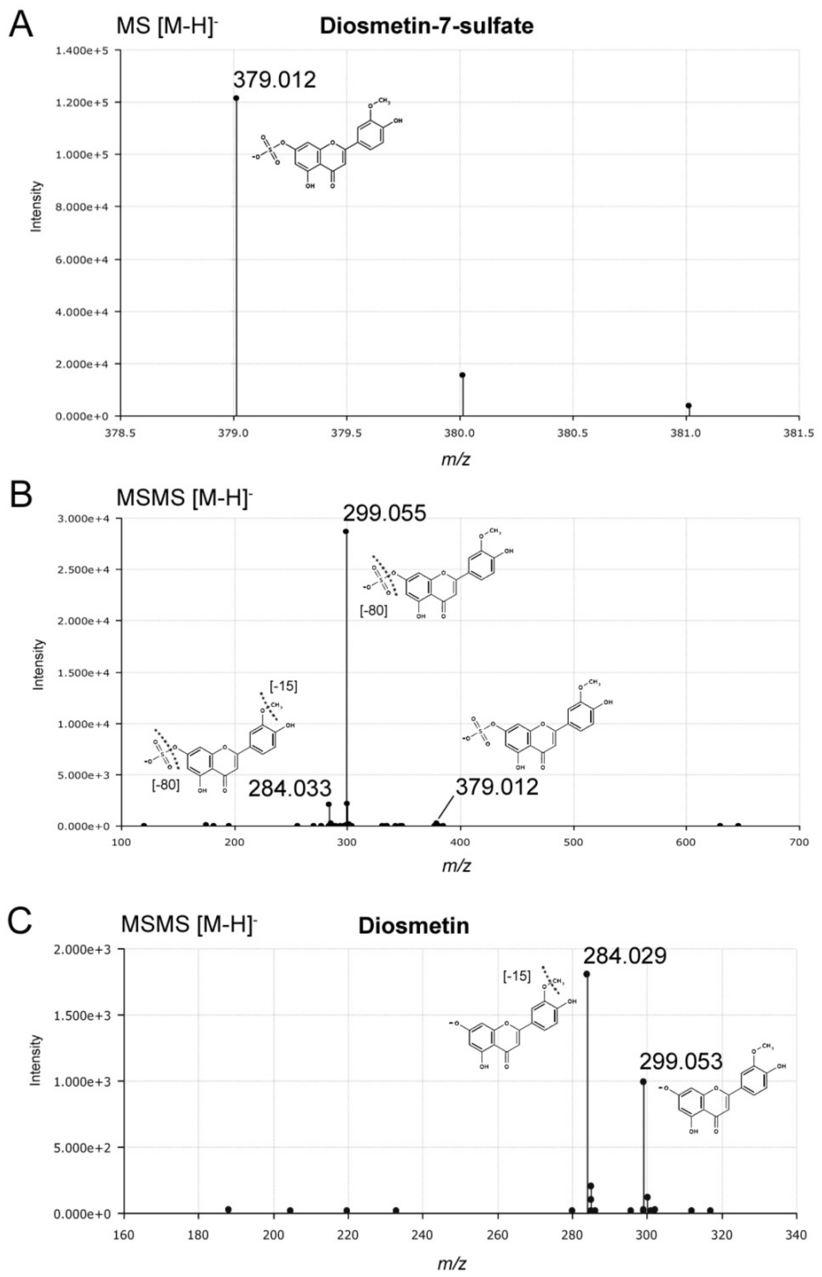

Figure S12. Targeted MS/MS identification of diosmetin-7-sulfate. (***A***) Diosmetin-7-sulfate (i.e. luteolin-3'-methylether-7-sulfate) was detected by UHPLC-QTOF-MS in negative ionization mode as molecular ion *m/z* $[M\text{-}H]^-$ 379.012 ($C_{16}H_{11}O_9S$) and its natural isotopic pattern distribution, at a retention time of 4.5 min. (***B***) MS/MS fragmentation (30-80 eV) of the molecular ion with the loss of sulfate moiety to form diosmetin ion *m/z* $[M\text{-}SO_3\text{-}H]^-$ 299.055 ($C_{16}H_{11}O_6$). A smaller fragment ion resulting from further loss of the methyl group ($-CH_3$) was detected at *m/z* $[M\text{-}H]^-$ 284.033 ($C_{15}H_8O_6$). The same fragmentation pattern was reported in previous analyses on *Z. marina* (see Table 1, main article). Diosmetin-7-sulfate is reported as luteolin-3'-methylether-7-sulfate in the METLIN database (ID: 49230) by UHPLC-QTOF-MS/MS experimental detection of the molecular ion *m/z* $[M\text{-}H]^-$ 379.012, and the predicted *in silico* fragmentation spectra in positive mode producing diosmetin ion at *m/z* $[M+H]^+$ 301.027. (***C***) Diosmetin molecular ion at *m/z* 299.053 ($C_{16}H_{11}O_6$) and detected MS/MS fragmentation with loss of the methyl group ($-CH_3$) detected at *m/z* $[M\text{-}H]^-$ 284.029 ($C_{15}H_8O_6$).

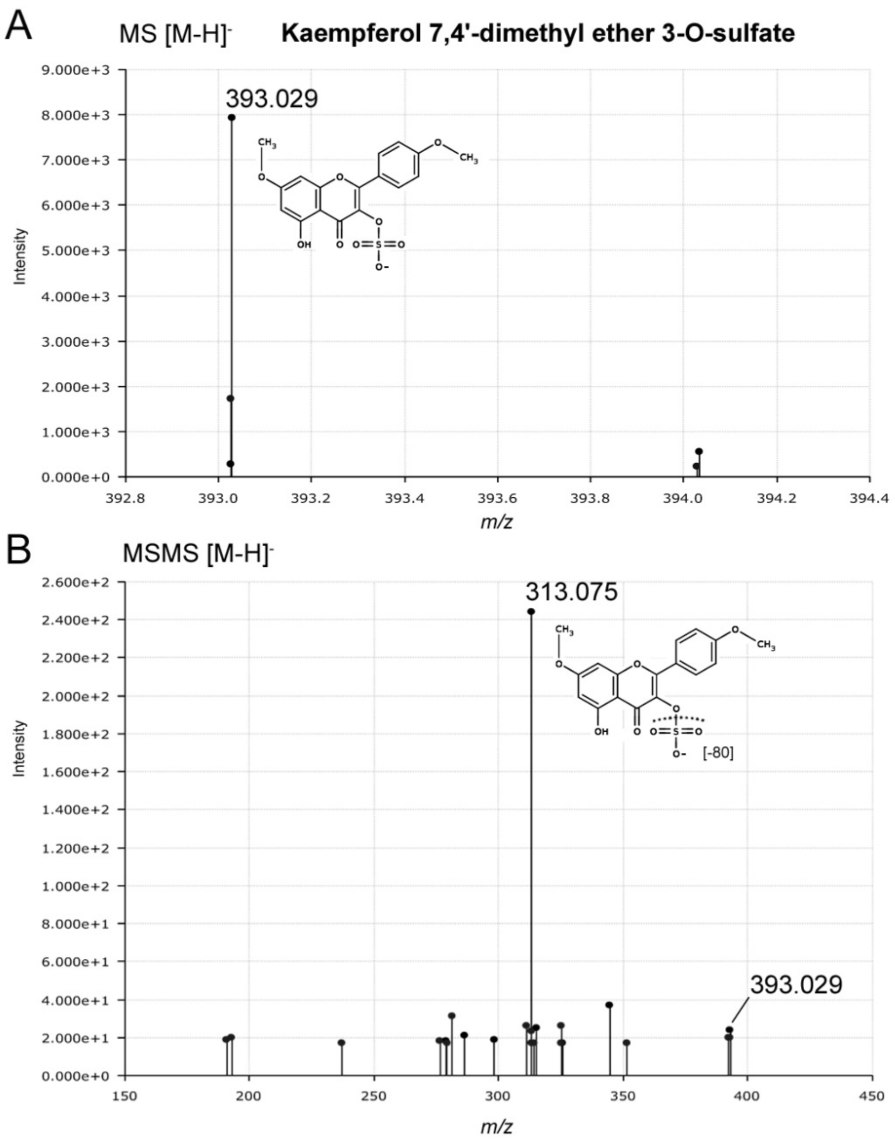

Figure S13. Targeted MS/MS identification of kaempferol-7,4'-dimethylether-3-O-sulfate (putative). (***A***) A compound previously not reported from eelgrass was detected here in *Z. marina* by UHPLC-QTOF-MS in negative ionization mode as ion *m/z* $[M-H]^-$ 393.029 ($C_{17}H_{13}O_9S$) at a retention time of 4.9 minutes. (***B***) MS/MS fragmentation (30-80eV) of the of the molecular ion with neutral loss of sulfate moiety to form kaempferol 7,4'-dimethylether ion at *m/z* $[M-SO_3-H]^-$ 313.075 ($C_{17}H_{13}O_6$). Kaempferol-7,4'-dimethylether-3-O-sulfate is similarly reported in the METLIN database (ID: 51011) for experimental detection with UHPLC-QTOF-MS/MS of the intact ion at *m/z* $[M-H]^-$ 393.029 and *in silico* fragmentation spectra in positive ionization mode with predicted loss of the sulfate moiety to produce the major ion of kaempferol-7,4'-dimethylether ion at *m/z* $[M+H]^+$ 315.085.

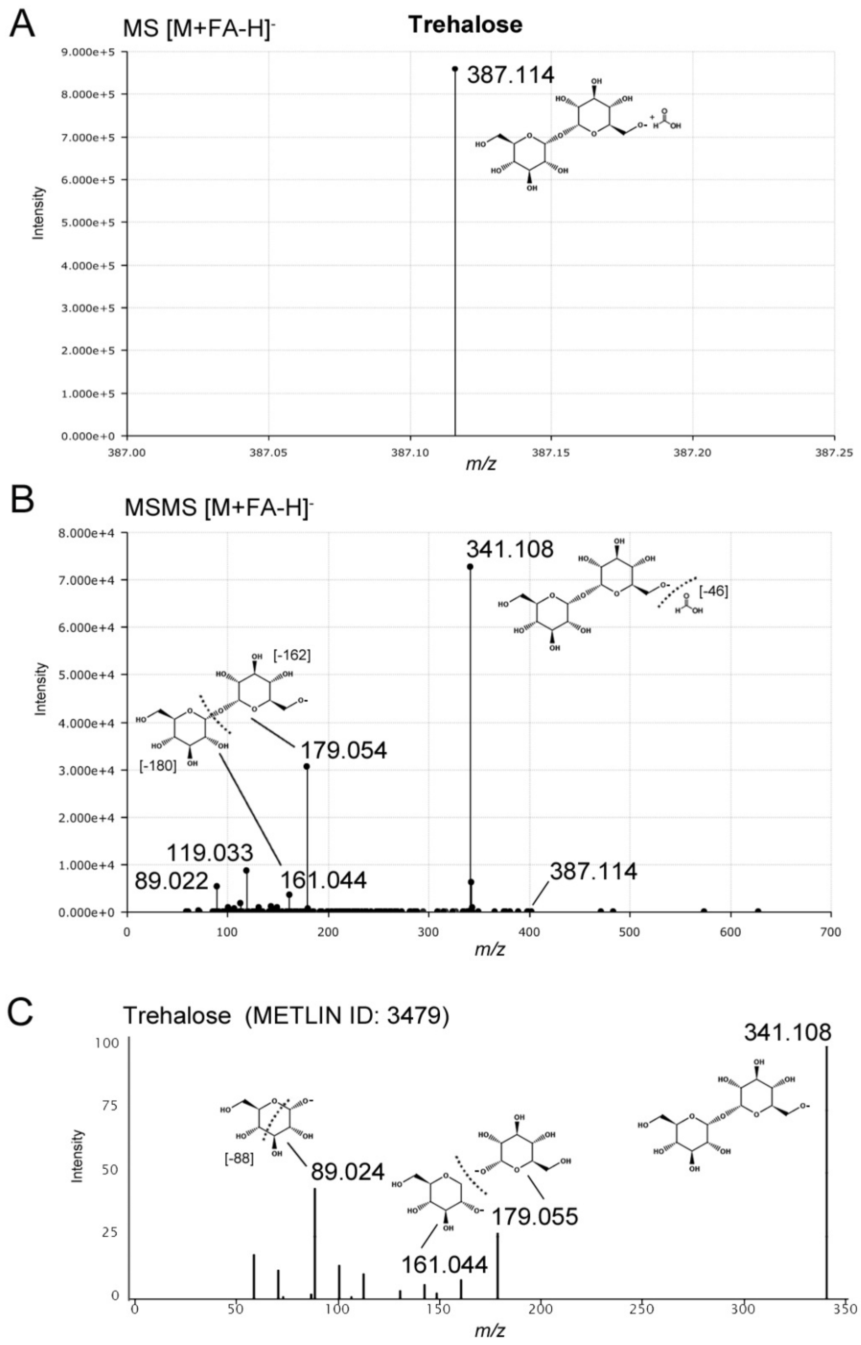

Figure S14. Targeted MS/MS identification of trehalose. (***A***) Trehalose was detected with UHPLC-QTOF-MS in negative ionization mode as formic acid (FA) adduct at *m/z* $[M+FA-H]^-$ 387.114 at a retention time of 0.6 min. (***B***) MS/MS fragmentation (30-80eV) resulted in the neutral loss of the formic acid adduct to form molecular ion *m/z* $[M-H]^-$ 341.108 ($C_{11}H_{21}O_{11}$) and two fragment ions from the cleavage of the disaccharide α-α bond forming D-glucopyranose ion at *m/z* $[M-H]^-$ 179.054 ($C_6H_{11}O_6$) and the other respective fragment ion with loss of water molecule at *m/z* $[M-H]^-$ 161.044 ($C_6H_9O_5$).(***C***) METLIN database reports the same fragmentation for trehalose (ID: 3479) with UHPLC-QTOF-MS/MS detection of the molecular ion *m/z* $[M-H]^-$ 341.108 and experimental fragmentation at *m/z* $[M-H]^-$ 179.055 and 161.044, and a smaller fragment was observed at *m/z* $[M-H]^-$ 89.024 ($C_3H_5O_3$) resulting from the cleavage of the hexose ring[11].

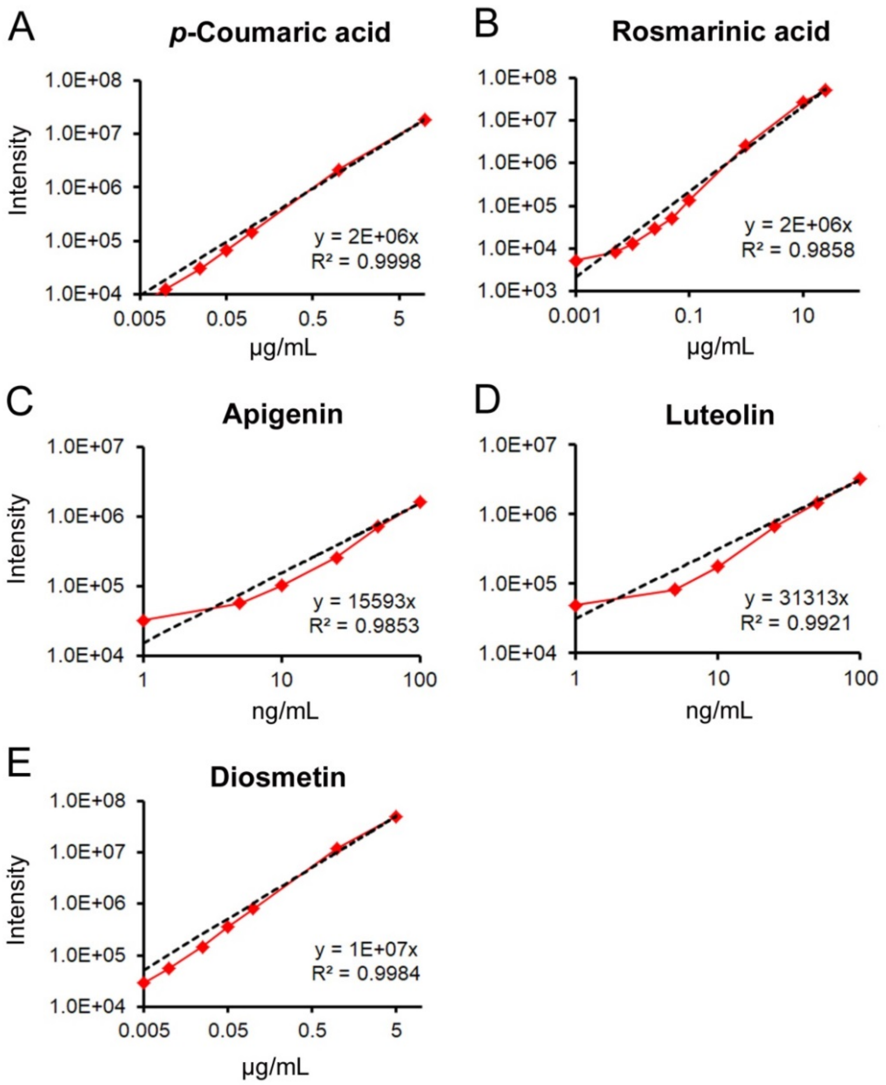

Figure S15. Quantification of phenolic compounds in eelgrass extracts by UHPLC-QTOF-MS. Phenolic compound concentrations were measured by comparing the peak intensity of compounds in eelgrass extracts with pure standards of (**A**) *p*-coumaric acid, (B) rosmarinic acid, (***C***) apigenin, (***D***) luteolin and (***E***) diosmetin measured at different concentrations (from 1 ng/mL to 25 µg/mL) depending on each compound´s relative abundance in the extract. Four technical replicates were injected in quadruplicate (1 µL) into the UHPLC-QTOF-MS system using a linear gradient: 99% A1 (0-7 min), 0% A1 (7-8 min) followed by column reconditioning to 11 min. The same flow rate (0.5 mL/min) and MS conditions used for analyzing the extracts were applied. Absolute concentrations in all eelgrass extracts were quantified fitting the integrated *m/z* peak intensity for each compound on the calibration curve, using *p*-coumaric acid for all the phenolic acids except for rosmarinic acid, and using the flavonoids for their respective sulfated forms.

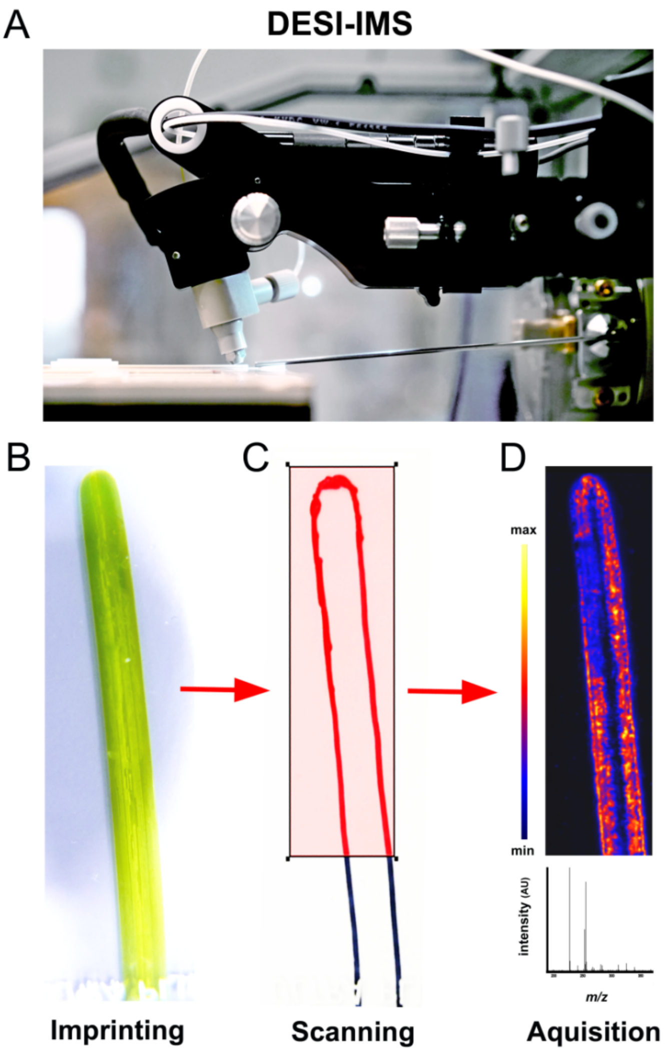

Figure S16. DESI-IMS workflow. (***A***) DESI-IMS instrument used in this study for analyzing eelgrass surface-associated metabolites. The DESI source was installed on the Xevo G2-XS qTOF-MS. (***B***) Young and healthy eelgrass leaf was imprinted between two clean glass slides (SuperFrost Ultra Plus®, Thermo Fisher Scientific) under a 5 kg weight for 24 h. (***C***) IMS was performed using an electrospray solvent mixture of methanol and water (95:5 v/v) at a constant flow rate of 1.5 µL min^{-1} and rastered over the selected surface area of the imprinted glass surface at a scan rate of 150 µm sec^{-1}. (***D***) Imaging data were acquired with the QTOF-MS in negative ionization mode at the *m/z* range $[M-H]^-$ 100-1500 and resolution of 150 µm (pixel size). All photos by Stefano Papazian.

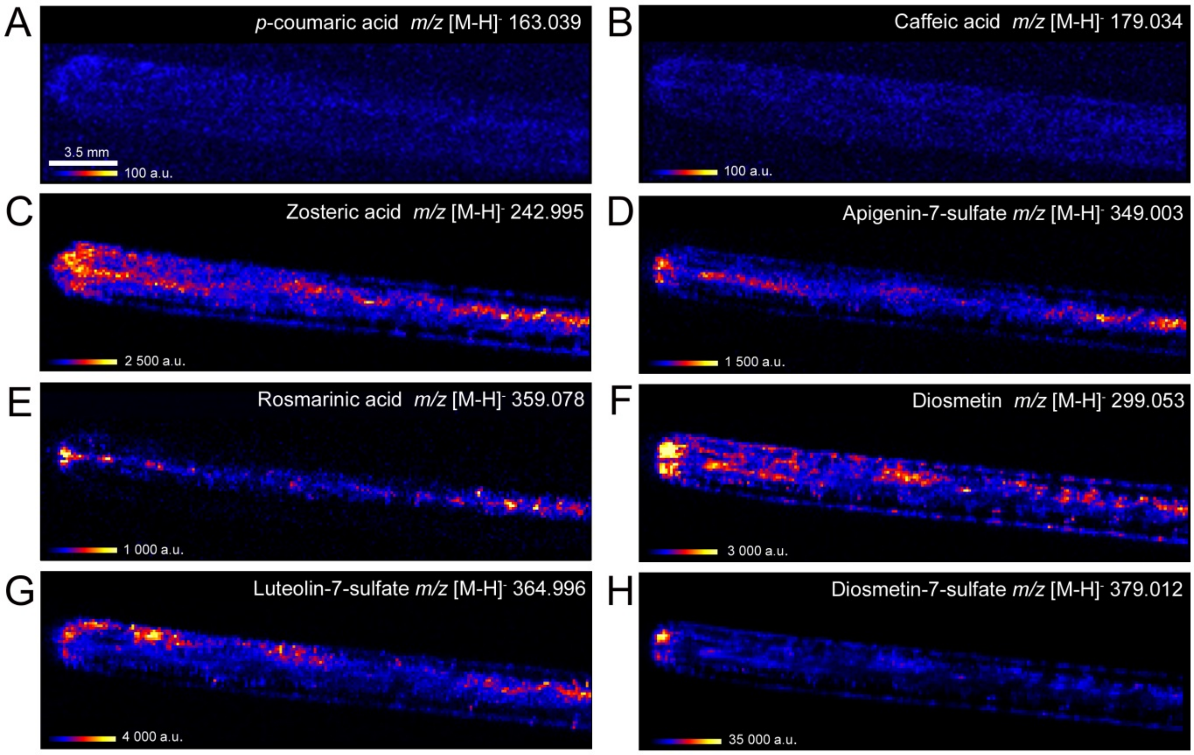

Figure S17. Surface-associated phenolic metabolites identified by DESI-IMS at 150-µm resolution on the eelgrass leaf surface. DESI-IMS images show the distribution and the relative intensity of *m/z* $[M-H]^-$ ions. Heat-map scaling shows the highest local accumulation points indicated by the respective maximum range on the intensity scale (a.u.). (***A***) *p*-coumaric acid, (***B***) caffeic acid, (***C***) zosteric acid (i.e. sulfated form of *p*-coumaric acid), (***D***) apigenin-7-sulfate, (***E***) rosmarinic acid, (***F***) diosmetin, (***G***) luteolin-7-sulfate, and (***H***) diosmetin-7-sulfate. Total scanned surface area = 312 mm^2 (3.1 cm^2). Actual scanned leaf surface = 125 mm^2 (1.25 cm^2). Scale bar = 3.5 mm.

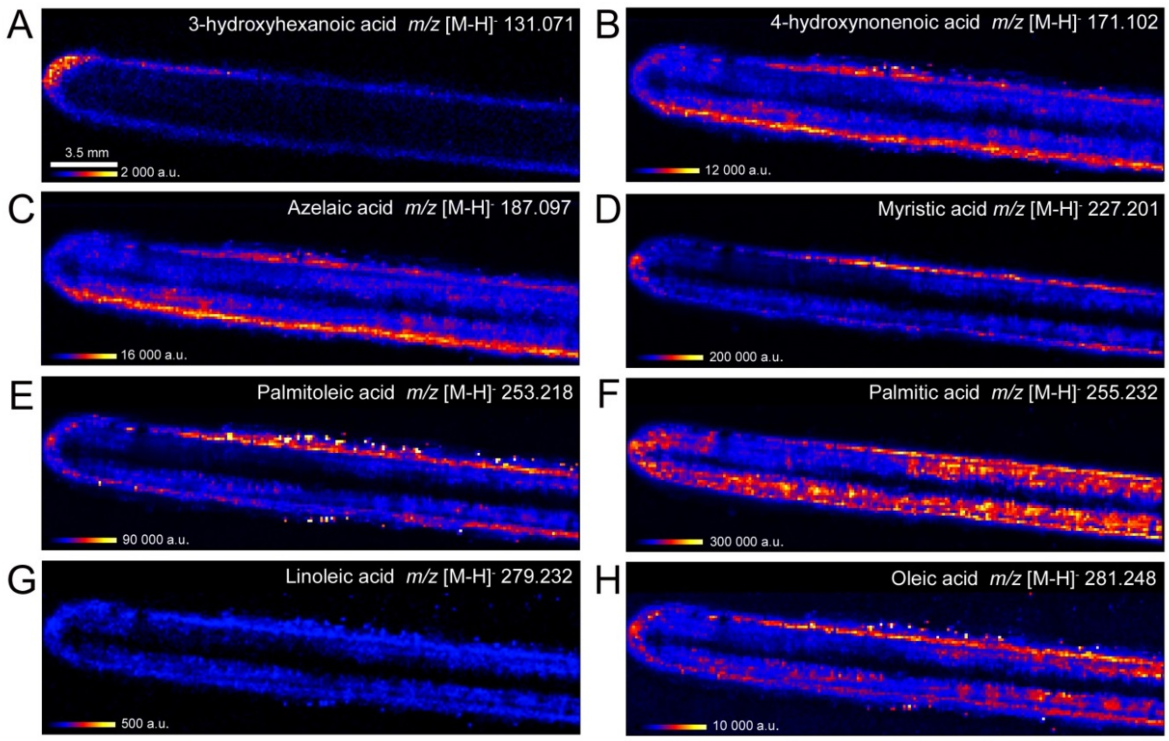

Figure S18. Surface-associated fatty acids and carboxylic acids identified by DESI-IMS at 150-µm resolution on the eelgrass leaf surface. DESI-IMS images show the distribution and the relative intensity of *m/z* $[M\text{-}H]^-$ ions. Heat-map scaling shows the highest local accumulation points indicated by the respective maximum range on the intensity scale (a.u.). Carboxylic acids (***A***) 3-hydroxyhexanoic acid, (***B***) 4-hydroxynonenoic acid, and (***C***) nonanedioic acid (i.e. azelaic acid); and fatty acids (***D***) myristic acid, (***E***) palmitoleic acid, (***F***) palmitic acid, (***G***) linoleic acid, and (***H***) oleic acid. Total scanned surface area = 312 mm^2 (3.1 cm^2). Actual scanned leaf surface = 125 mm^2 (1.25 cm^2). Scale bar = 3.5 mm.

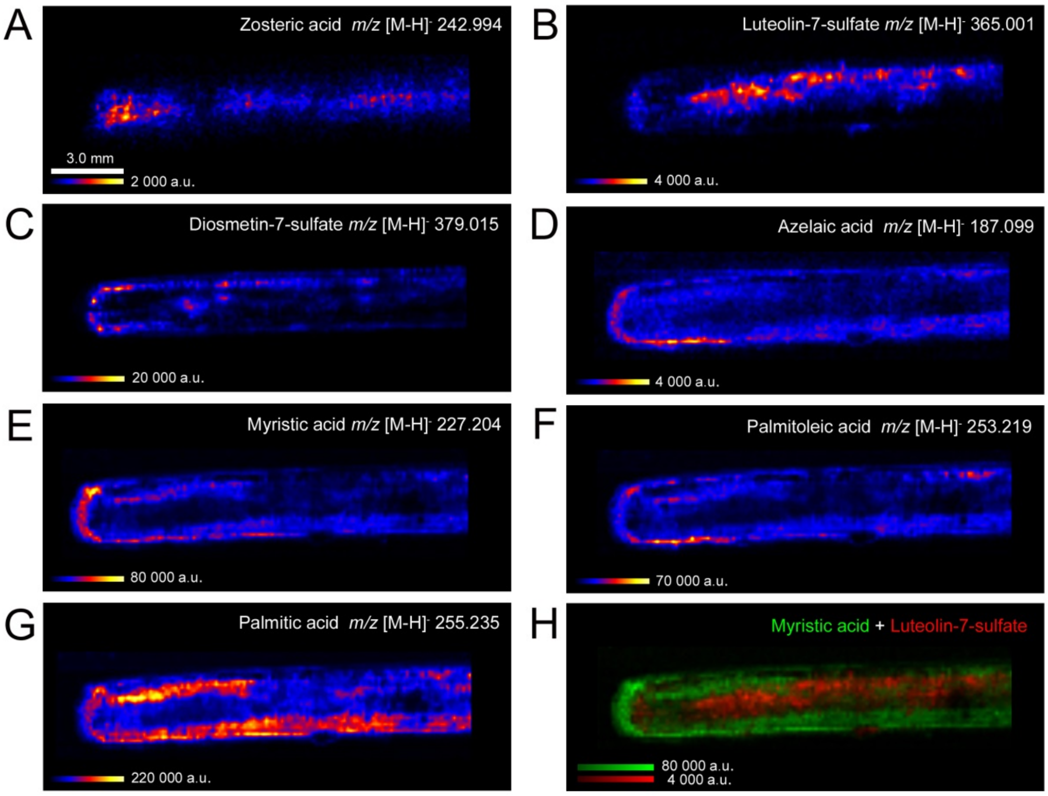

Figure S19. Surface-associated phenolic metabolites and fatty acids identified by DESI-IMS at 150-μm resolution on the leaf surface from a second eelgrass replicate. DESI-IMS images show the distribution and the relative intensity of *m/z* $[M\text{-}H]^-$ ions. Heat-map scaling shows the highest local accumulation points indicated by the respective maximum range on the intensity scale (a.u.). (***A-C***) phenolic compounds: zosteric acid, luteolin-7-sulfate, and diosmetin-7-sulfate; and (***D-G***) fatty acids: azelaic acid (peroxide derivative), myristic, palmitoleic, and palmitic acid. (***H***) Superimposition of myristic acid (green) and luteolin-7-sulfate (red). Total scanned surface area = 145 mm^2 (1.45 cm^2). Actual scanned leaf surface area = 63.8 mm^2 (0.65 cm^2). Scale bar = 3.0 mm.

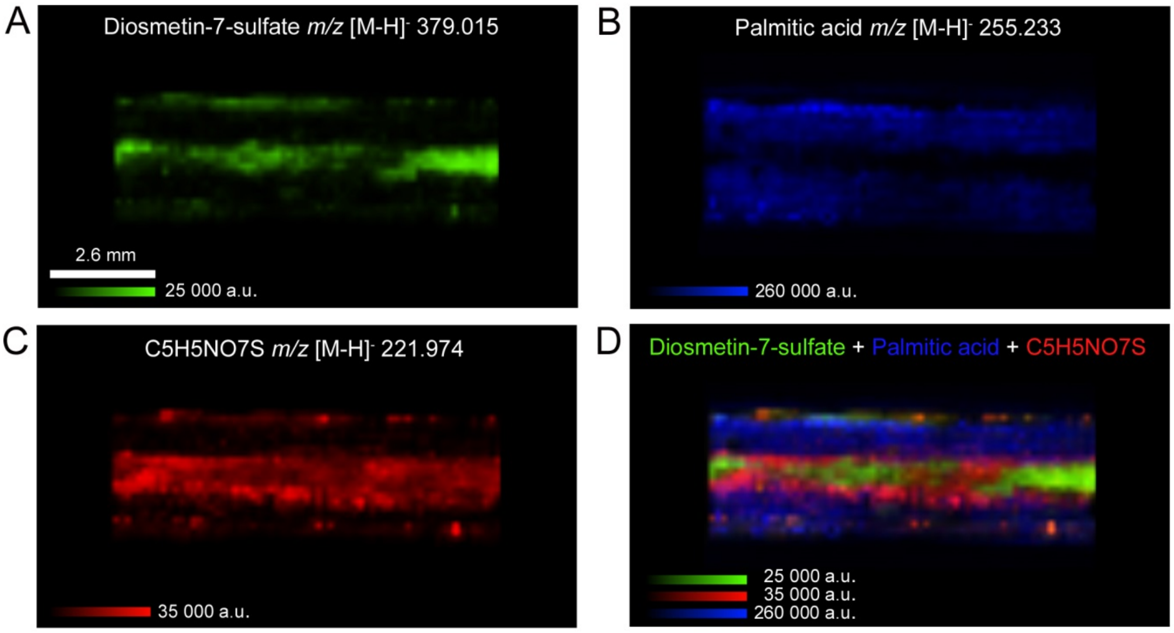

Figure S20. Surface-associated phenolic metabolites and fatty acids identified by DESI-IMS at 150-µm resolution on the leaf surface in a third replicate of the eelgrass, DESI-IMS images show the relative intensity of *m/z* $[M\text{-}H]^-$ ions and their distribution towards the lower lamina regions along the midvein and lateral veins. (***A***) Diosmetin-7-sulfate, (***B***) palmitic acid, and (***C***) unknown compound of putative molecular formula $C_5H_5NO_7S$, as suggested by SIRIUS. No match in METLIN has been found for this metabolite within the *m/z* window of 10 ppm. For the corresponding *m/z* $[M+H]^+$ ion at a Δ of 6.6 ppm, the Dictionary of Natural Products (DNP) database reports the formula C_9H_6BrNO (possibly 4-bromoacetyl-benzonitrile) from several sea sponges, tunicates, and *Acinetobacter* sp. (***D***) Superimposition of these three compounds, diosmetin-7-sulfate (green), palmitic acid (blue), and putative $C_5H_5NO_7S$ (red). Total scanned surface area = 71.2 mm^2 (0.71 cm^2). Actual scanned leaf surface = 41.9 mm^2 (0.42 cm^2). Scale bar = 2.6 mm.

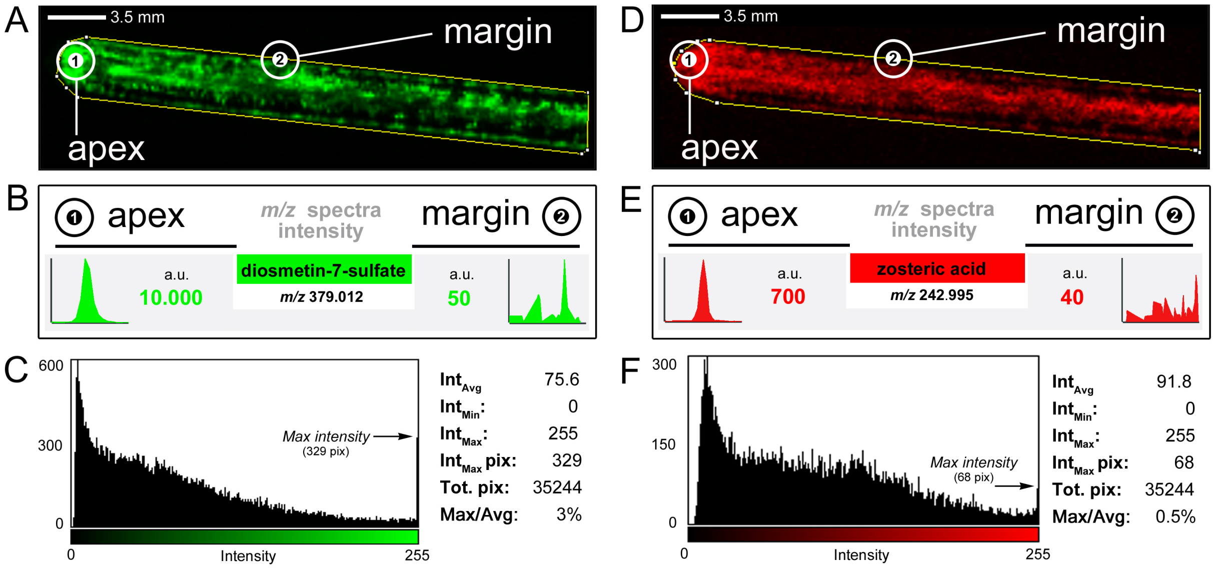

Figure S21. Local maxima concentrations of the phenolic compounds on the eelgrass leaf surface. DESI-IMS scan and spectra inspected in OpenMSI and ImageJ analyses are shown in (***A-C***) for the ion *m/z* $[M\text{-}H]^-$ 379.012 corresponding to diosmetin-7-sulfate (DS), and in (***D-F***) for the ion *m/z* $[M\text{-}H]^-$ 242.995 corresponding to zosteric acid (ZA). Scanned leaf surface area = 125 mm^2 (1.25 cm^2). Size-bar = 3.5 mm. OpenMSI spectra showed differential distribution and accumulation on the surface of DS and ZA (***B,E***) with 20 to 200-fold change between patches of local maxima around the apex and the lowest intensities around the leaf margins. Analyses of the histogram distribution in ImageJ for the two compounds (***C,F***) shows the total pixel count at each intensity level across the scanned leaf surface, with the maximum (Int_{max}) and average (Int_{avg}) pixel intensity representing a concentration maxima of 3% for DS, and 0.5% for ZA per surface area (Int_{max}*pix/Int_{avg}*pix).

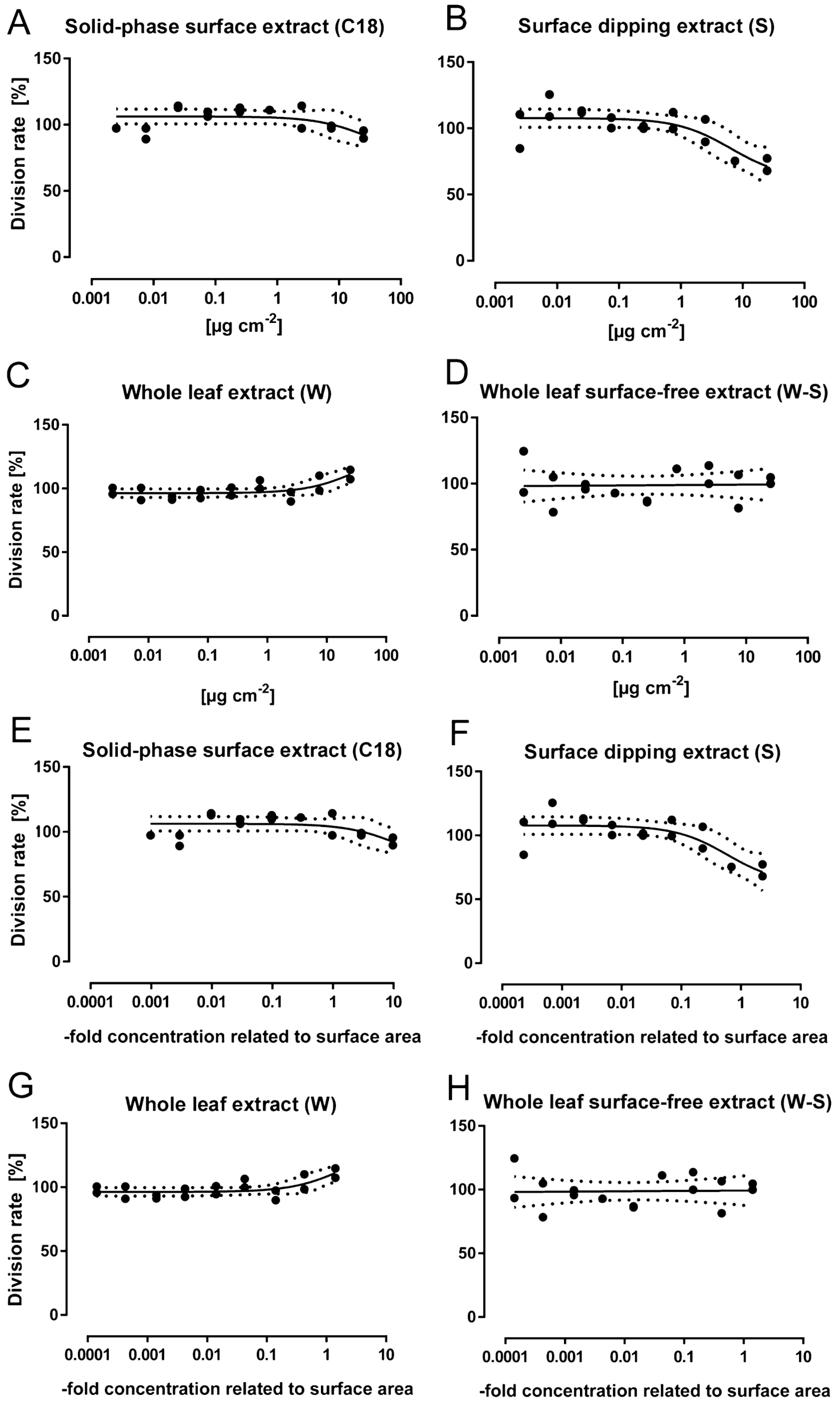

Figure S22. Bioactivity of eelgrass extracts on the growth of *Cryptococcus fonsecae*. Extracts of *Z. marina* obtained by solid-phase surface adsorption (C18), surface dipping (S), whole leaf (W), and whole leaf after surface dipping (W-S), were tested for their effect on the growth of the marine yeast *C. fonsecae*, a facilitator of the wasting disease in *Z. marina*. (***A-D***) The inhibition or activation effect on the yeast growth relative to controls is reported as percentage (%) of cell division rate for the respective extract concentrations (µg.cm^{-2}), and as (***E-H***) natural concentration related to the leaf surface area or the whole leaf.

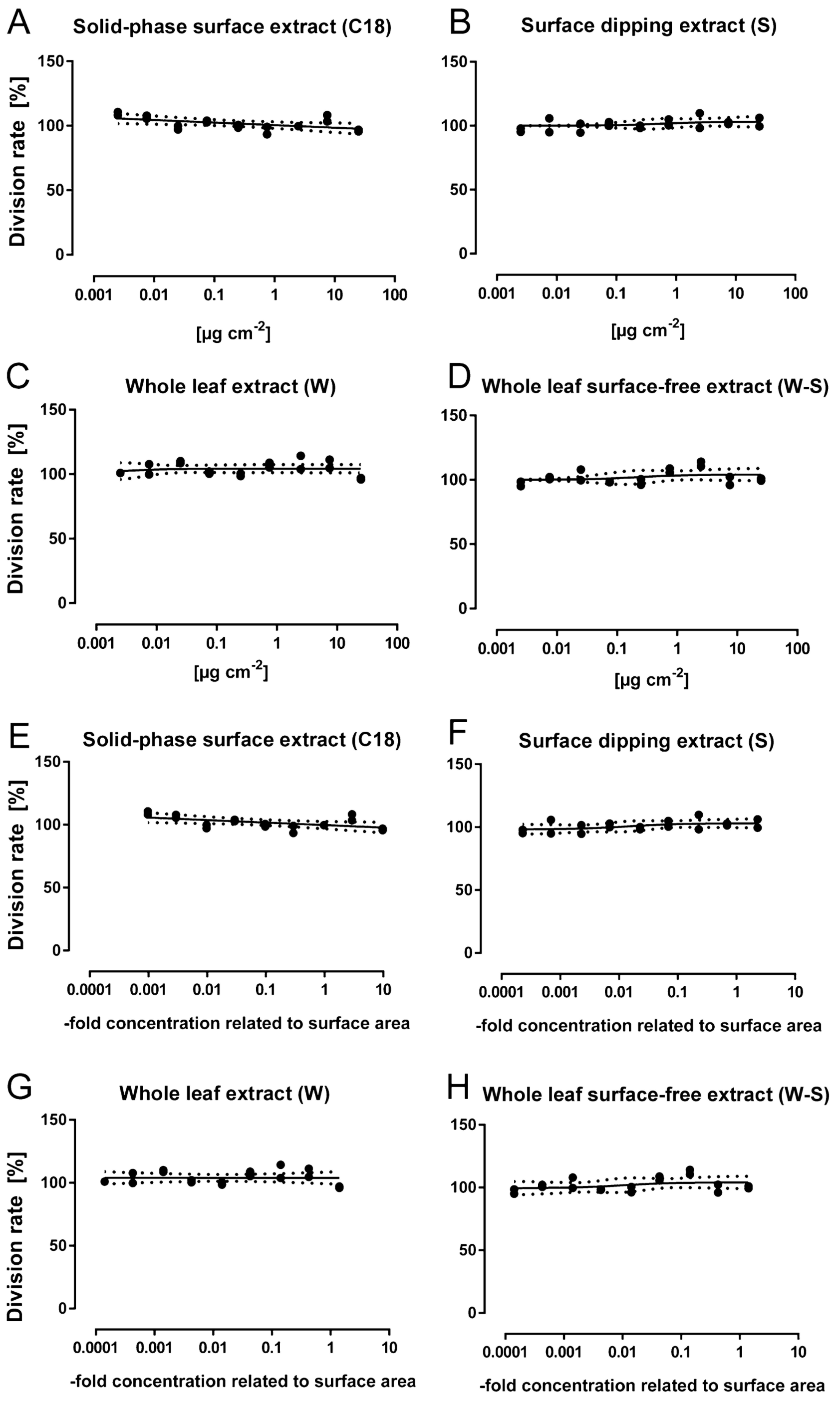

Figure S23. Bioactivity of eelgrass extracts on the growth of *Debaryomyces hansenii.* Extracts of *Z. marina* obtained by solid-phase surface adsorption (C18), surface dipping (S), whole leaf (W), and whole leaf after surface dipping (W-S), were tested for their effect on the growth of the marine epiphytic yeast *D. hansenii,* previously isolated from *Z. marina.* ***(A-D)*** The inhibition or activation effect on the yeast growth relative to controls is reported as percentage (%) of cell division rate for the respective extract concentrations (μg.cm^{-2}), and as (***E-H***) natural concentration related to the leaf surface area or the whole leaf.

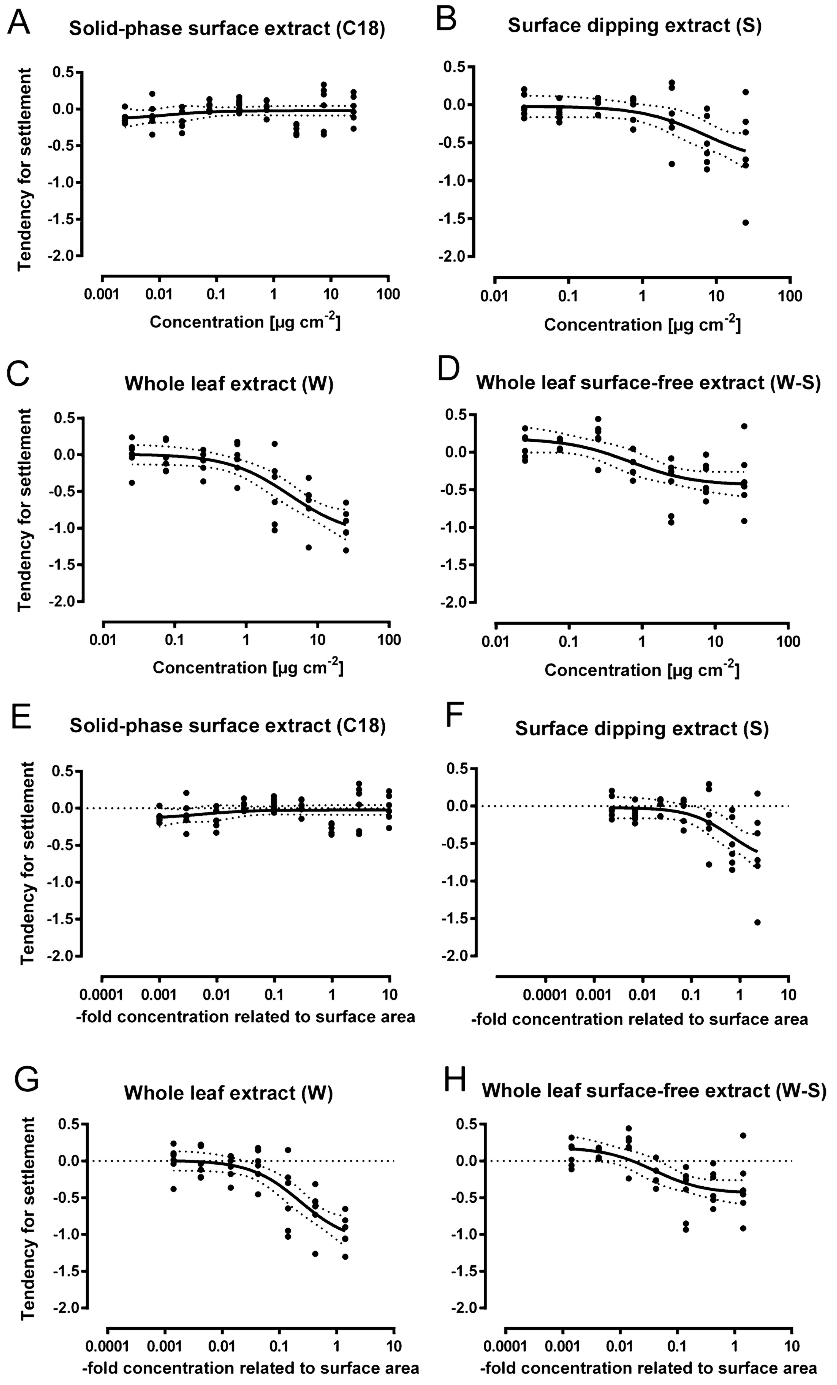

Figure S24. Bioactivity of eelgrass extracts on the settlement of *Debaryomyces hansenii.* Extracts of *Z. marina* obtained by solid-phase surface adsorption (C18), surface dipping (S), whole leaf (W), and whole leaf after surface dipping (W-S), were tested for their effect on the settlement of the marine epiphyte yeast *D. hansenii,* previously isolated from *Z. marina. (**A-D**)* The inhibition effect on yeast settlement relative to controls is reported for the respective extract concentrations (µg.cm^{-2}), and as (***E-H***) natural concentration related to the leaf surface area or the whole leaf.

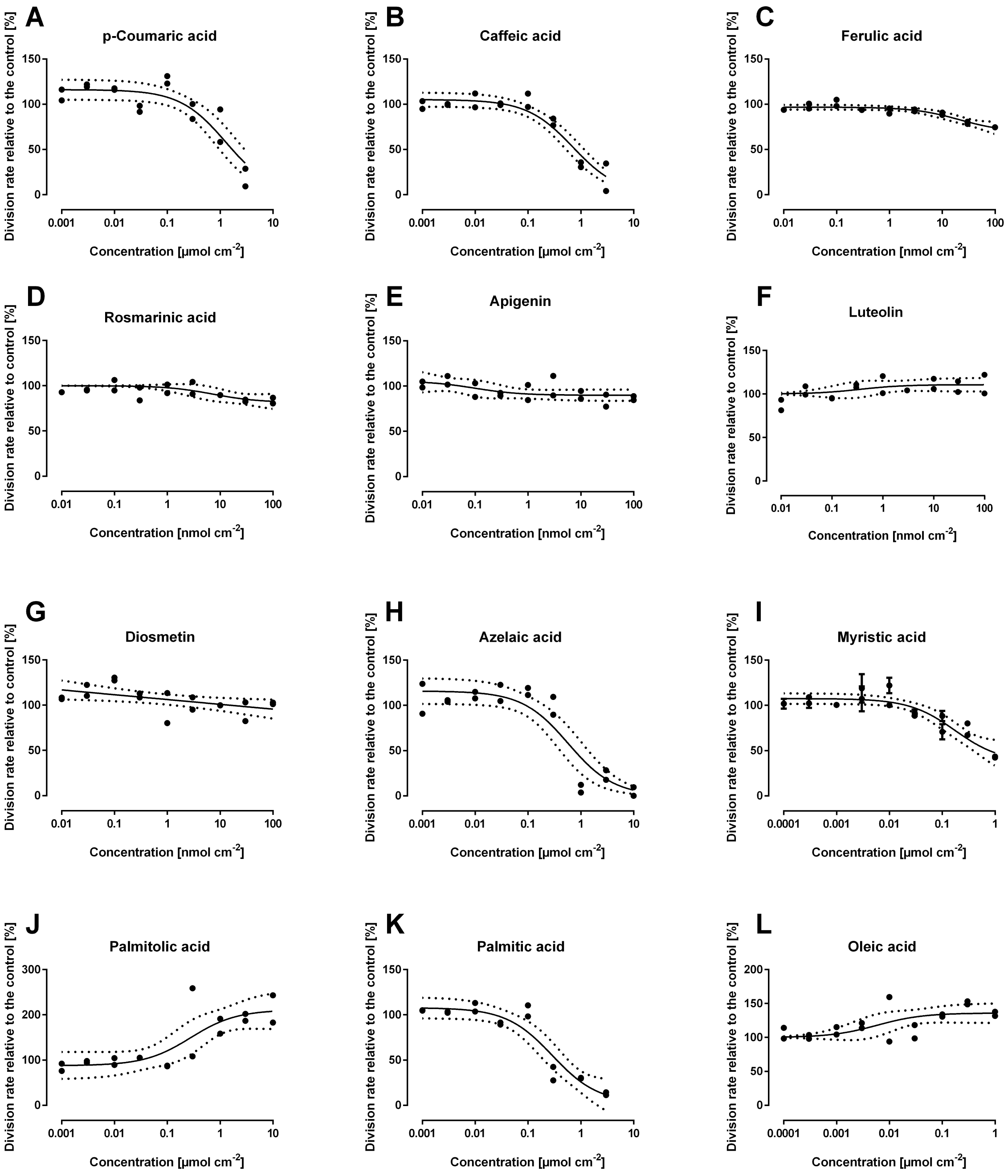

Figure S25. Bioactivity of eelgrass surface-associated metabolites on the growth of the marine yeast *Cryptococcus fonsecae*. Pure compounds identified on eelgrass leaf surfaces were tested for their effect on the growth of the epiphytic yeast *C. fonsecae*, a facilitator of wasting disease in *Z. marina.* (***A-G***) Effects of desulfated phenolic acids and flavones. (***H-L***) Effects of azelaic acid and fatty acids. For each pure compound the inhibition or activation effect on the yeast growth is reported as percentage (%) of cell division rate at the respective concentration range (nmol or µmol.cm^{-2}) relative to the control.

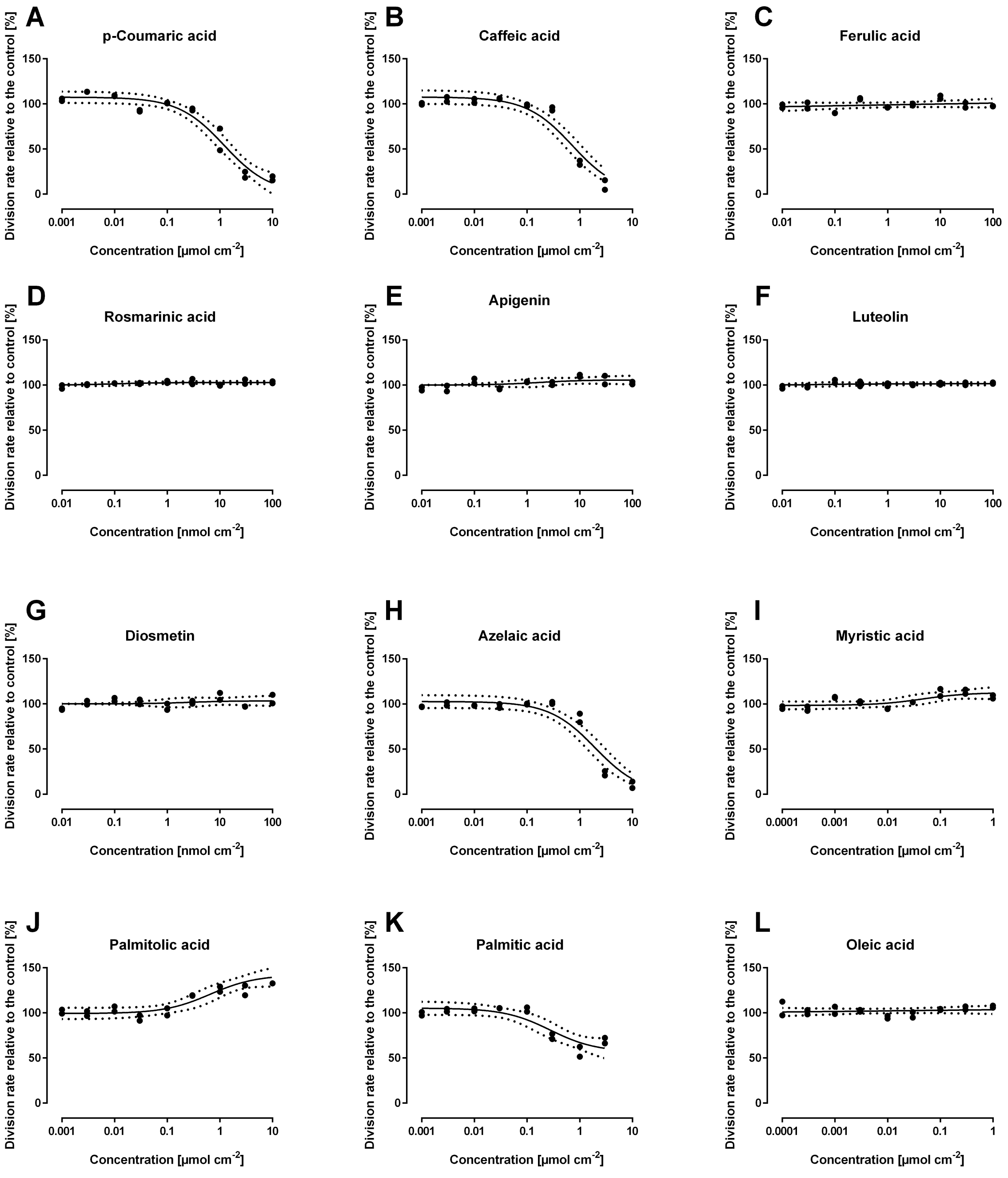

Figure S26. Bioactivity of eelgrass surface-associated metabolites on the growth of the marine yeast *Debaryomyces hansenii*. Pure compounds identified on eelgrass leaf surfaces were tested for their effect on the growth of *D. hansenii* strain previously isolated from *Z. marina*. (***A-G***) Effects of desulfated phenolic acids and flavones. (***H-L***) Effects of azelaic acid and fatty acids. For each pure compound the inhibition or activation effect on the yeast growth is reported as percentage (%) of cell division rate at the respective concentration range (nmol or µmol.cm^{-2}) relative to the control.

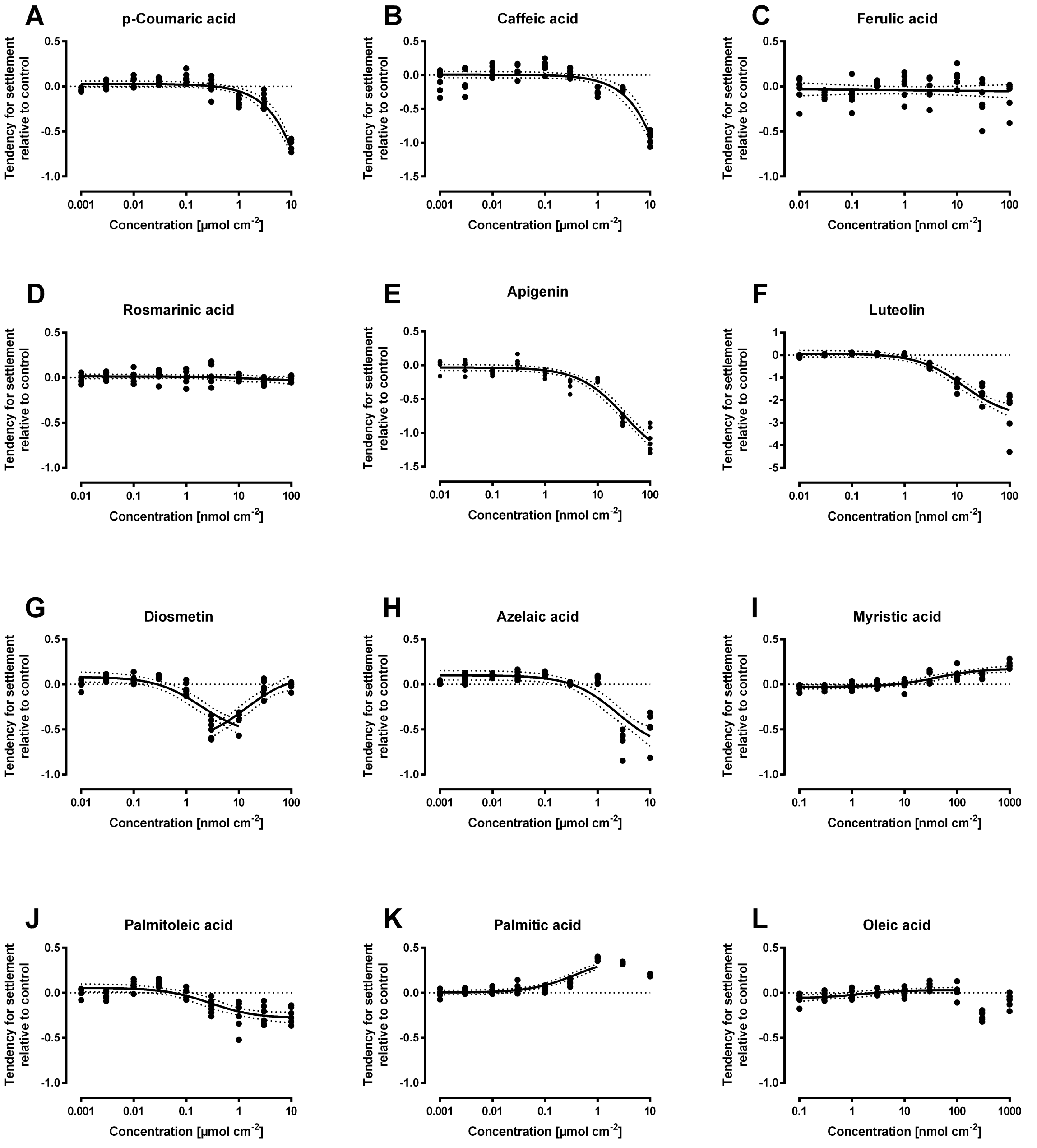

Figure S27. Bioactivity of eelgrass surface-associated metabolites on the settlement of the marine yeast *Debaryomyces hansenii*. Pure compounds of metabolites identified on eelgrass leaf surfaces were tested for their effect on the settlement of *D. hansenii* strain previously isolated from *Z. marina*. (***A-G***) Effects of (desulfated) phenolic acids and flavones. ***(H-L***) Effects of azelaic acid and fatty acids. For each pure compound the inhibition or activation effect on the yeast settlement is reported at the respective concentration range (nmol or µmol.cm^{-2}) relative to the control.

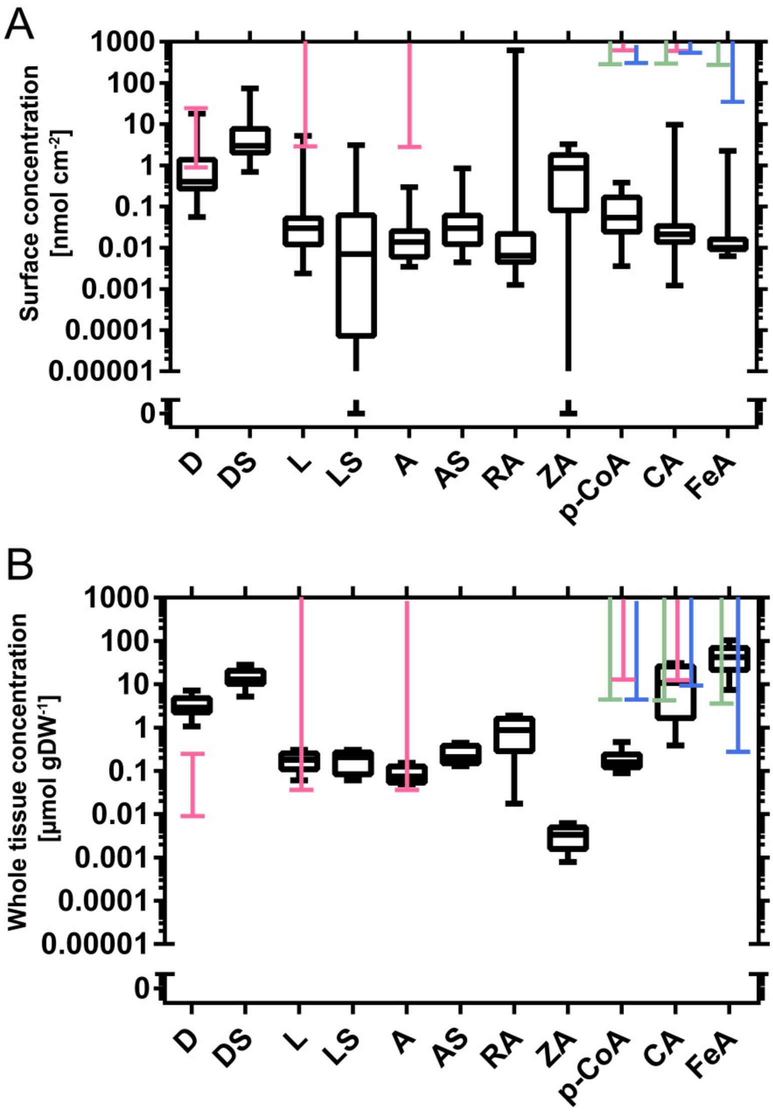

Figure S28. Antifouling activity of pure phenolic compounds relative to concentrations quantified on (***A***) total leaf surface (nmol.cm^{-2}), and in (***B***) whole leaf tissue (µmol.gDW^{-1}); see also Table S1. Concentrations are shown as boxplots indicating median +/- quartiles and compared to the bioactivity concentration ranges tested in the bioassays for the respective compounds, showing a significant inhibition of the settlement of *D. hansenii* (pink), of the growth of *D. hansenii* (green), or of the growth of *C. fonsecae* (blue); see also in Figs. S25-S27. Compound abbreviations: diosmetin (D), diosmetin-7-sulfate (DS), apigenin (A), apigenin-7-sulfate (AS), luteolin (L), luteolin-7-sulfate (LS), rosmarinic acid (RA), zosteric acid (ZA), *p*-coumaric acid (*p*-CoA), caffeic acid (CA), and ferulic acid (FeA).

Tables

Table S1. Concentrations of phenolic compounds in the eelgrass extracts per leaf surface area or leaf tissue dry weight and relative surface concentration maxima.

	p-Co	CA	FeA	RA	ZA	A	L	D	AS	LS	DS
**Surface extracts** (nmol.cm^{-2}), LC-MS											
**C18**	>0.01	>0.01	>0.01	>0.01	n.d.	>0.01	>0.01	0.06	>0.01	>0.01	0.69
**S** (avg.)	0.09 ±0.03	0.05 ± 0.02	0.15 ± 0.12	1.85 ±1.64	1.16 ±0.33	0.02 ±0.01	0.07 ±0.04	1.26 ±0.63	0.05 ±0.02	0.08 ±0.06	6.41 ±2.66
**S9**(rep.)*	0.34	0.25	1.24	16.59	3.26	0.07	0.45	6.82	0.19	0.64	29.57
**S10**(rep.)*	0.38	9.64	2.25	618.8	1.78	0.29	5.18	17.96	0.84	3.08	73.36
**Whole leaf extracts** (µmol gDW^{-1}), LC-MS											
**W** (avg.)	0.20 ±0.03	13.33 ±3.49	46.38 ±9.62	0.92 ±0.20	0.003 ±0.005	0.09 ±0.01	0.18 ±0.03	3.53 ±0.54	0.25 ±0.04	0.19 ±0.02	15.00 ±2.11
**Surface local maxima** (µmol cm^{-2}), DESI-IMS											
**Imprint**	>0.001	0.001	n.d.	0.078	0.232	0.004	0.004	0.042	0.003	0.006	0.211

Concentrations of phenolic compounds (± standard error) detected in eelgrass leaf surfaces and whole leaf tissues were measured comparing UHPLC-QTOF-MS integrated peak areas of each compound in the extracts versus the responses of calibration curves obtained from pure phenolic compounds. Surface concentration maxima were calculated comparing accumulation patterns of each compound from DESI-IMS images versus the average total intensity across the leaf surface (see Figure S21). Surface areas were calculated with the assumptions of a dry weight (DW) to fresh weight (FW) ratio of 1:10 and a surface area of 79 cm² corresponding to 1 g FW tissues in *Z. marina*. Compound abbreviations: *p*-coumaric acid (*p*-Co), caffeic acid (CA), ferulic acid (FeA), rosmarinic acid (RA), zosteric acid (ZA), apigenin (A), luteolin (L), diosmetin (D), apigenin-7-sulfate (AS), luteolin-7-sulfate (LS), and diosmetin-7-sulfate (DS). *Two surface dipping (S) replicates displayed the highest concentrations for all the phenolics and particularly RA. Chemical variation was possibly due to differences in extraction efficiency but also to actual biological variation among eelgrass specimens, as suggested by the different ratios between different compounds.

Table S2. Molecular networking and MS/MS spectral dereplication in GNPS.[6]
See also the corresponding molecular network in the main article (Figure 1).

m/z [M-H]⁻	Rt (min)	Library ID match	Molecular formula
**Cluster I**			
452.232	7.4	Lyso-PE(16:0/0:0)	C21H44NO7P
474.215	6.7	PE(18:3(6Z,9Z,12Z)/0:0)	C23H41NO7P
480.263	7.5	PE(18:0/0:0)	C23H48NO7P
481.211	7.6	PE(18:0/0:0)	C23H48NO7P
502.246	6.8	Lyso-PC (15:0/0:0) [M-CH3]⁻	C26H48NO7P
505.211	7.0	Lyso-PE(0:0/20:2(11Z,14Z))	C25H48NO7P
529.219	6.3	PG(20:5(5Z,8Z,11Z,14Z,17Z)/0:0)	C26H43O9P
530.222	6.3	PC(19:3(10Z,13Z,16Z)/0:0)	C27H50NO7P
540.285	7.5	PC(20:5(5Z,8Z,11Z,14Z,17Z)/0:0)	C28H48NO7P
559.268	7.3	PG(22:4(7Z,10Z,13Z,16Z)/0:0)	C28H49O9P
562.268	6.8	PC(18:1(9E)/2:0)	C28H54NO8P
564.285	7.2	PC(18:0/2:0)	C28H56NO8P
712.451	8.9	PE(16:0/18:3(9Z,12Z,15Z))	C39H72NO8P
714.466	8.7	PC(13:0/18:2(9Z,12Z))	C39H74NO8P
721.323	6.7	PG(16:0/16:0)	C38H75O10P
741.431	7.5	PG(16:1(9Z)/18:3(6Z,9Z,12Z))	C40H71O10P
742.434	8.1	PE(20:2(11Z,14Z)/16:0)	C41H78NO8P
745.457	7.0	PG(15:1(9Z)/19:1(9Z))	C40H75O10P
745.458	7.5	PG(15:1(9Z)/19:1(9Z))	C40H75O10P
792.459	10.1	PE(18:3(9Z,12Z,15Z)/22:2(13Z,16Z))	C45H80NO8P
801.510	8.8	PG(16:1(9Z)/22:1(11Z))	C44H83O10P
819.490	10.5	PG(18:1/22:6)	C46H77O10P
821.496	10.7	PG(18:0/22:6)	C46H79O10P
826.523	8.7	PE(20:1(11Z)/22:1(11Z))	C47H90NO8P
**Cluster II**			
365.106	4.3	Luteolin-7-sulfate*	C15H10O9S
379.109	4.5	Diosmetin-7-sulfate*	C16H12O9S
407.140	9.3	PA(16:1(9Z)/0:0)	C19H37O7P
673.316	8.1	PA (16:0/18:1(11Z))	C37H71O8P
**Cluster III**			
491.051	5	Sophoraisoflavanone D	C30H36O6
671.098	5.2	Sagerinic acid [M-H-CH2O2]	C36H32O16
**Cluster IV**			
387.068	0.6	Sophoricoside [M-H-CO2]	C21H20O10

PA: Phosphatidic acid; PC: Phosphatidylcholine; PE: Glycerophosphoethanolamine; PG: Glycerophosphoglycerol. * Manually annotated MS/MS nodes for sulfated flavonoids.

Table S3. Putative molecular formulae for unknown compounds predicted *in-silico* by computing MS/MS fragmentation trees (SIRIUS)[9].

m/z [M-H]⁻	Putative formula candidates (top 3)		
	1	2	3
112.982	C4H3O2P	H6NO2P2	-
118.925	CHNP3	-	-
173.080	C4H10N6O2	C6H12N3O3	C2H8N9O
174.954	C4O8	C3HN2O5P	H3NO8P
183.010	C11H4O3	C3H10N2O3P2	C5H5N4O2P
190.927	C3H4N2P4	CH9OP5	C7HNP3
193.049	C6H6N6O2	C4H11N4O3P	C8H8N3O3
206.970	C6HN4O3P	C7N2O6	C2H4N5O3P2
207.010	C13H4O3	C2H4N6O6	C12H5N2P
215.128	C7H16N6O2	C11H20O4	C12H16N4
221.972	C5H7NO7S	C7H2N3O4P	C6H3N5OP2
228.958	C2H2N2O11	C6H4N2O4P2	C5H5N4OP3
265.147	C16H18N4	C15H22O4	C9H23N4O3P
266.150	C13H21N3O3	C9H17N9O	C7H22N7O2P
267.144	C11H24O7	C8H16N10O	C12H20N4O3
269.044	C11H6N6O3	C8H15O8P	C10H10N2O7
281.121	C7H18N6O6	C8H14N10O2	C18H18O3
284.031	C13H7N3O5	C7H8N7O4P	C6H12N3O8P
285.039	C11H6N6O4	C10H10N2O8	C8H15O9P
289.090	C8H14N6O6	C19H14O3	C7H18N2O10
293.177	C11H27N4O3P	C15H24N3O3	C17H26O4
294.181	C15H25N3O3	C11H21N9O	C9H26N7O2P
295.137	C8H20N6O6	C19H20O3	C9H16N10O2
297.152	C19H22O3	C8H22N6O6	C9H18N10O2
298.155	C17H21N3O2	C12H28O6P	C13H17N9
300.058	C7H16N3O8P	C12H9N6O4	C6H10N10O3P
309.150	C20H22O3	C14H23N4O2P	C9H22N6O6
309.172	C18H22N4O	C17H26O5	C16H27N2O2P
309.173	C18H22N4O	C17H26O5	C16H27N2O2P
311.168	C9H24N6O6	C10H20N10O2	C13H28O8
311.306	C19H40N2O	C17H38N5	-
312.171	C18H23N3O2	C14H19N9	C13H30O6P
313.167	C17H22N4O2	C16H26O6	C15H27N2O3P
315.180	C12H24N6O4	C13H20N10	C17H24N4O2
321.211	C20H26N4	C9H26N10O3	C18H31N2OP
323.168	C21H24O3	C10H24N6O6	C15H25N4O2P
325.184	C10H26N6O6	C21H26O3	C11H22N10O2
326.187	C19H25N3O2	C15H21N9	C14H32O6P
327.163	C13H28O9	C21H20N4	C20H24O4
329.233	C14H30N6O3	C18H34O5	C19H30N4O
337.203	C20H26N4O	C19H30O5	C18H31N2O2P
338.027	C12H9N3O9	C13H5N7O5	C8H5N9O7
339.198	C22H28O3	C11H28N6O6	C16H29N4O2P
339.199	C22H28O3	C12H24N10O2	C11H28N6O6
340.203	C16H23N9	C20H27N3O2	C15H27N5O4
341.178	C14H30O9	C22H22N4	C21H26O4
347.188	C10H24N10O4	C6H20N16O2	C7H26N9O7
351.218	C20H32O5	C21H28N4O	C14H33N4O4P
353.143	C10H22N6O8	C11H18N10O4	C14H26O10
353.199	C20H26N4O2	C18H31N2O3P	C19H30O6
355.159	C11H20N10O4	C10H24N6O8	C14H28O10
365.140	C22H22O5	C18H18N6O3	C16H23N4O4P
377.143	C23H22O5	C9H14N16O2	C11H26N2O12
381.175	C12H26N6O8	C13H22N10O4	C24H22N4O
381.230	C21H34O6	C22H30N4O2	C18H26N10

383.187	C23H28O5	C19H24N6O3	C24H24N4O
387.115	C11H22N3O12	C12H18N7O8	C9H20N6O11
388.290	C14H35N11O2	C18H39N5O4	C10H31N17
391.282	C19H40N2O6	C20H36N6O2	C16H32N12
393.171	C13H26N6O8	C14H22N10O4	C24H26O5
397.159	C18H26N2O8	C15H18N12O2	C30H22O
407.187	C14H28N6O8	C25H28O5	C15H24N10O4
455.247	C14H34N9O8	C13H28N16O3	C19H42N2O6P2
458.920	C14H5O16P	C6H11N2O16P3	C8H6N4O15P2
465.305	C20H38N10O3	C19H42N6O7	C31H38N4
465.305	C25H38N8O	C24H42N4O5	C23H46O9
477.305	C32H38N4	C20H42N6O7	C31H42O4
481.258	C22H43O9P	C18H39N6O7P	C30H34N4O2
481.258	C24H38N2O8	C18H39N6O7P	C22H43O9P
483.273	C20H36N8O6	C24H40N2O8	C18H41N6O7P
491.100	C16H16N10O9	C22H16N6O8	C15H20N6O13
502.293	C28H37N7O2	C23H37N9O4	C24H33N13
505.114	C17H18N10O9	C23H18N6O8	C24H14N10O4
505.258	C20H39N6O7P	C26H38N2O8	C28H30N10
506.261	C24H37N5O7	C22H42N3O8P	C29H37N3O5
507.274	C26H40N2O8	C24H45O9P	C20H41N6O7P
527.254	C28H32N8O3	C27H36N4O7	C26H40O11
529.266	C21H42N2O13	C22H38N6O9	C26H42O11
531.275	C25H32N12O2	C18H36N12O7	C19H32N16O3
531.282	C22H40N6O9	C26H44O11	C21H44N2O13
533.288	C13H38N14O9	C25H34N12O2	C19H34N16O3
540.332	C27H47N3O8	C28H43N7O4	C24H39N13O2
554.250	C19H33N13O7	C29H37N3O8	C24H37N5O10
559.314	C28H48O11	C24H44N6O9	C22H49N4O10P
562.316	C23H45N7O9	C31H37N11	C30H41N7O4
563.320	C32H44N4O5	C31H48O9	C28H40N10O3
564.330	C24H47N5O10	C25H43N9O6	C26H39N13O2
577.257	C25H42N2O13	C23H30N16O3	C38H34N4O2
577.272	C36H38N2O5	C20H38N10O10	C21H34N14O6
609.135	C16H31N6O17P	C19H22N12O12	C34H26O11
617.296	C26H34N16O3	C25H38N12O7	C39H42N2O5
625.137	C23H26N6O15	C22H30N2O19	C24H22N10O11
626.140	C22H21N13O10	C21H25N9O14	C28H21N9O9
627.302	C22H40N14O8	C29H36N14O3	C28H40N10O7
643.333	C25H40N16O5	C24H44N12O9	C38H48N2O7
645.330	C26H42N14O6	C41H46N2O5	C25H46N10O10
646.331	C34H45N7O6	C29H45N9O8	C25H41N15O6
671.141	C26H20N14O9	C25H24N10O13	C20H24N12O15
673.361	C28H46N14O6	C27H50N10O10	C33H50N6O9
677.063	C18H18N10O19	C29H18N4O16	C25H14N10O14
721.367	C27H46N16O8	C36H50N8O8	C31H50N10O10
737.450	C35H62N8O9	C34H66N4O13	C32H54N18O3
744.495	C36H59N17O	C35H63N13O5	C34H67N9O9
745.408	C38H58N4O11	C37H58N6O10	C34H50N16O4
761.456	C43H58N10O3	C42H62N6O7	C41H66N2O11
763.468	C41H68N2O11	C39H56N16O	C54H60N4
791.498	C41H60N16O	C40H64N12O5	C39H68N8O9
793.515	C41H62N16O	C40H66N12O5	C36H62N18O3
807.494	C41H60N16O2	C40H64N12O6	C39H68N8O10
819.531	C46H72N6O7	C45H76N2O11	C42H68N12O5
825.551	C40H70N14O5	C39H74N10O9	C36H66N20O3
826.564	C43H69N15O2	C38H69N17O4	C33H69N19O6
829.478	C43H58N16O2	C58H62N4O	C42H62N12O6
835.525	C43H64N16O2	C42H68N12O6	C41H72N8O10
845.418	C36H62N8O15	C34H50N22O5	C49H54N10O4
860.651	C44H79N17O	C39H79N19O3	C40H79N17O4

869.560	C49H74N8O6	C40H70N16O6	C50H74N6O7
870.564	C49H69N13O2	C40H65N21O2	C37H73N15O9
873.452	C42H70N2O17	C40H58N16O7	C55H62N4O6
883.542	C54H72N6O5	C51H68N10O4	C39H68N18O6
955.557	C37H72N20O10	C52H76N8O9	C51H80N4O13
959.600	C51H80N10O8	C50H84N6O12	C48H72N20O2
983.598	C52H86N6O13	C52H84N6O12	C50H72N20O2
997.582	C58H78N8O7	C42H78N16O12	C55H70N18O
998.526	C60H65N13O2	C55H65N15O4	C61H65N11O3

Table S4. Principle component multivariate statistics (PCA) for eelgrass extracts analyzed with UHPLC-QTOF-MS, see Figure 1*C* in the main article.

Component	R2X	$R2X_{(cum)}$	Eigenvalue	Q2	Limit	$Q2_{(cum)}$	Sign.
0	Cent.						
1	0.221	0.221	7.31	0.078	0.03	0.078	R1
2	0.197	0.418	6.5	0.102	0.03	0.172	NS
3	0.116	0.534	3.83	-0.011	0.03	0.163	R1

Table S5. Supervised multivariate statistics (PLS-DA) for eelgrass extracts analyzed with UHPLC-QTOF-MS, see Supplementary Figure S7.

Component	R2X	$R2X_{(cum)}$	Eigenvalue	R2Y	$R2Y_{(cum)}$	Q2	Limit	$Q2_{(cum)}$	Sign.
0	Cent.								
1	0.213	0.213	7.01	0.322	0.322	0.257	0.05	0.257	R1
2	0.175	0.387	5.76	0.193	0.515	-0.0591	0.05	0.213	NS
3	0.0781	0.465	2.58	0.179	0.694	0.222	0.05	0.388	R1

Table S6. Multivariate statistics for metabolite distribution on the eelgrass surfaces analyzed with DESI-IMS (PCA), see Figure 3*E* in the main article.

Component	R2X	$R2X_{(cum)}$	Eigenvalue	Q2	Limit	$Q2_{(cum)}$	Sign.
0	Cent.						
1	0.72	0.72	28.8	0.601	0.036	0.601	R1
2	0.145	0.865	5.82	0.417	0.036	0.767	R1
3	0.052	0.917	2.07	0.265	0.037	0.829	R1

Table S7. Bioactivity of *Z. marina* extracts and individual surface-associated metabolites on the growth of the marine epiphytic yeast *C. fonsecae.*

Growth of *C. fonsecae*					
	**Bioactivity**	**Effect (%)**	**Best fit R^2**	**EC_{50} [µg/cm^2]**	**95% CI**
Extracts					
C18	Inhibition	25%	0.289	33.2	0.005 - 204286
S	Inhibition	40%	0.646	6.45	0.720 - 57.8
W	Activation	30%	0.487	34.5	0.153 - 7809
W-S	None		0.001		
Compounds					
Apigenin	Inhibition	20%	0.558	5.7	0.55 - 58.4
Luteolin	None		0.308		
Diosmetin	None		0.291		
Rosmarinic acid	Inhibition	20%	0.340	7.3	0.73 - 73.2
p-Coumaric acid	Inhibition	80%	0.813	1280	679 -2411
Caffeic acid	Inhibition	90%	0.913	708	448 - 1119
Ferulic acid	Inhibition	30%	0.831	21	5 - 76
Myristic acid	Inhibition	65%	0.767	174.4	65 - 468
Oleic acid	Activation	35%	0.389	5.8	0.67 - 50.8
Azelaic acid	Inhibition	100%	0.859	150.5	276 - 1198
Palmitic acid	Inhibition	98%	0.899	288.5	112 - 744
Palmitoleic acid	Activation	110%	0.679	275.7	46.5 - 1633

EC_{50} values were estimated based on dose-response studies fitting logistic functions to datasets. For several compounds, maximal responses could not be observed, which is reflected in large EC_{50} confidence intervals.

Table S8. Bioactivity of *Z. marina* extracts and surface-associated metabolites on the growth of the marine epiphytic yeast *D. hansenii.*

	**Growth of *D. hansenii***				
	**Bioactivity**	**Effect (%)**	**Best fit R^2**	**EC_{50} [µg/cm^2]**	**95% CI**
Extracts					
C18	None		0.284		
S	None		0.165		
W	None		0.024		
W-S	None		0.121		
Compounds					
Apigenin	None		0.258		
Luteolin	None		0.126		
Diosmetin	None		0.075		
Rosmarinic acid	None		0.279		
p-Coumaric acid	Inhibition	100%	0.950	1208	655 - 2228
Caffeic acid	Inhibition	100%	0.928	726	471 - 1120
Ferulic acid	None		0.062		
Myristic acid	Activation	10%	0.522	45.6	3.8 - 554
Oleic acid	None		0.498		
Azelaic acid	Inhibition	100%	0.914	1967.0	1243 - 3113
Palmitic acid	Inhibition	50%	0.826	274.2	75 - 997
Palmitoleic acid	Activation	40%	0.804	654.0	179 - 2392

EC_{50} values were estimated based on dose-response studies fitting logistic functions to datasets. For several compounds, maximal responses could not be observed, which is reflected in large EC_{50} confidence intervals.

Table S9. Bioactivity of the *Z. marina* extracts and surface-associated metabolites on the settlement of the marine epiphytic yeast *D. hansenii.*

Settlement of *D. hansenii*					
	**Bioactivity**	**Effect (%)**	**Best fit R^2**	**EC_{50} [µg/cm^2]**	**95% CI**
Extracts					
C18	None		0.042		
S	Inhibition	80%	0.345	7.088	0.7413 - 67.7
W	Inhibition	90%	0.651	4.096	1.323 - 12.7
W-S	Inhibition	60%	0.438	0.6953	0.1309 - 3.7
Compounds					
Apigenin	Inhibition	96%	0.910	32.9	20.6 - 52.7
Luteolin	Inhibition	100%	0.863	13.0	7.6 - 22.3
Diosmetin (lower)*	Inhibition	75%	0.773	1.9	0.8 - 4.5
Diosmetin (upper)	Inhibition	75%	0.809	13.6	4.5 - 41.6
Rosmarinic acid	None		0.052		
p-Coumaric acid	Inhibition	50 %	0.861	> 10000	-
Caffeic acid	Inhibition	90 %	0.825	> 1000	-
Ferulic acid	None		0.002		
Myristic acid	Activation	50%	0.686	39.4	16.0 - 96.9
Oleic acid	Inhibition	15%	0.284	1.4	0.13 - 14.4
Azelaic acid	Inhibition	90%	0.748	2249.0	984 - 5137
Palmitic acid	Activation	150%	0.808	379.9	257 - 560
Palmitoleic acid	Inhibition	50%	0.655	279.8	107 - 731

EC_{50} values were estimated based on dose-response experiments fitting logistic functions to datasets. For several compounds, maximal responses could not be observed, which is reflected in large EC_{50} confidence intervals. *For the inhibitory effect of diosmetin observed at the tested concentration range, two EC_{50} values are calculated at the respective lower and upper limits of the curve (see also Figure S27).

Table S10. Blasting of *AZELAIC ACID INDUCED 1* (*AZI1*) on SeagrassDB[12]

Blasting of *Arabidopsis thaliana AZI1* full-length coding sequence (CDS) from the TAIR database (AT4G12470) to the *Z. marina* genome using the dedicated marine database SegrassDB (http://115.146.91.129/index.php)[12], produced a significant alignment score (77.0 bits, E-value = 7e-14) with the locus ZA008351 encoding for a lipid transfer protein that may possibly represent a similar function.

***Arabidopsis thaliana* locus AT4G12470 (*AZI1*):**

TAIR annotation:https://www.arabidopsis.org/servlets/TairObject?accession=locus:2135595

Encodes AZI1 (AZELAIC ACID INDUCED 1). Involved in the priming of salicylic acid induction and systemic immunity triggered by pathogen or azelaic acid. Targeting if AZI1 to chloroplasts is increased during SAR induction and that localization requires the PRR domain. It is involved in the uptake and movement of the azelaic acid signal.

ATGGCTTCAAAGAACTCAGCCTCTCTTGCTCTTTTCTTTGCGCTCAACATCCTCTTTTTCACCTTAACCG
TTGCAACAAATTGCAACTGCAAGCCAAGTCCTAAACCAAAGCCAGTCCCAAGTCCTAAGCCCAAGCCG
GTCCAATGTCCTCCTCCACCCCGTCCTTCAGTCCCAAGTCCTAATCCTAGGCCGGTCACACCTCCACG
CACCCCTGGTTCATCCGGAAACAGCTGTCCTATTGATGCTCTCAAGCTCGGTGTATGTGCAAATGTCTT
AAGCAGTCTACTCAACATCCAGTTGGGACAGCCATCCTCTCAACAATGTTGCTCGCTCATCCAAGGTTT
GGTTGACGTCGACGCTGCGATTTGTCTATGCACTGCTCTGAGGGCTAACGTTCTTGGTATCAACCTTA
ACGTTCCGATATCTCTCAGCGTTCTTCTCAACGTTTGTAACAGAAAGCTTCCATCTGGTTTCCAATGTG
CTTGA

***Zostera marina* locus ZA008351:**

Alignment >lcl|ZA008351
Length=480, Score = 77.0 bits (84), Expect = 7e-14
Identities = 98/135 (73%), Gaps = 0/135 (0%), Strand=Plus/Plus

BLAST / GO annotation for the locus ZA008351 in *Z. marina*http://115.146.91.129/annotation.php?ID=ZA008351
(uncharacterized lipid transfer protein)

ATGGCTTCCAAATCCATTTCCGCCGCTACTCTATTCATCCTTGCCACCTTCCTCCTCTTCTCAATTACCA
TGGCTTCTGCTGCTTGCCTTCCAAAATACAAAAAACCCAAGAGGCATACTCCAACCTATTCTCGCCCAG
TAGTACCATCCATTCCAAAATACTATCCTCCCAAAACCCCAGGCTGTTCAACCCCTACAATCCCAAGAA
CAGACTACCAAAAATGCCCAATCGATGCATTGAAGCTCAATGTGTGCGCCAATGTGCTTAATGGACTG
GTGAACGCTGTCATCGGAACTGAGGGTTCTTCTAAACCATGCTGCTCGCTCATTAAAGGTCTGGTAGA
TCTTGACGCCGCCGTCTGTCTTTGCACTGCTATCAAGGCCAATATCTTGGGCATCAATCTAAACTTGCC
TGTTTCTCTCAGCTTGCTCGTCAACCAGTGTGGAAGGGTCGTTCCTTCGCATTTCCAGTGCTCTTAA

Olsen et al., 2016
A0A0K9PBG0_ZOSMR GenomeNet:
http://www.genome.jp/dbget-bin/www_bget?uniprot:A0A0K9PBG0_ZOSMR

Protein sequence:

MASKSISVAT LFILATFLLF SITMASASCL PKYKKPKRHT PTYSRPVVPS IPKYYPPKTPGCSTPTIPRT
DYQKCPIDAL KLNVCANVLN GLVNAVIGTE GSSKPCCSLI KGLVDLDAAVCLCTAIKANI LGINLNLPVS
LSLLVNQCGR VVPSHFQCS

References

1. Röst, H.L., *et al.* OpenMS: A flexible open-source software platform for mass spectrometry data analysis. *Nat. Methods.* **13**, 741-748 (2016).
2. Pluskal, T., Castillo, S., Villar-Briones, A., Orešič, M. MZmine 2: Modular framework for processing, visualizing, and analyzing mass spectrometry-based molecular profile data. *BMC Bioinformatics* **11**, 395 (2010).
3. Rübel, O. *et al.* OpenMSI: A high-performance web-based platform for mass spectrometry imaging. *Anal. Chem.* **85**, 10354-10361 (2013).
4. Smith, C.A. et al. METLIN: A metabolite mass spectral database. *Therapeutic Drug Monitoring* **27**, 747-51 (2005).
5. Zhu, Z.J. *et al.* Liquid chromatography quadrupole time-of-flight mass spectrometry characterization of metabolites guided by the METLIN database. *Nat. Protoc.* **8**, 451-60 (2013).
6. Wang, M. et al. Sharing and community curation of mass spectrometry data with Global Natural Products Social Molecular Networking. *Nat. Biotechnol.* **34**, 828-837 (2016).
7. Chambers MC, *et al.* A cross-platform toolkit for mass spectrometry and proteomics. *Nat. Biotechnol.* **30**, 918-920 (2012).
8. Shannon, P. *et al.* Cytoscape: A software Environment for integrated models of biomolecular interaction networks. *Genome Res.* **3**, 2498-504 (2003).
9. Rasche, F., Svatoš, A., Maddula, R.K., Böttcher, C. & Böcker, S. Computing fragmentation trees from tandem mass spectrometry data. *Anal. Chem.* **83**, 1243-1251 (2011).
10. Weinberger, F. *et al.* Apoplastic oxidation of L-asparagine is involved in the control of the green algal endophyte *Acrochaete operculata* Correa & Nielsen by the red seaweed *Chondrus crispus* Stackhouse. *J. Exp. Bot.* **56**, 1317-26 (2005).
11. Calvano, C.D. *et al.* Structural characterization of neutral saccharides by negative ion MALDI mass spectrometry using a superbasic proton sponge as deprotonating matrix. *J. Am. Soc. Mass. Spectrom.* **28**, 1666-1675 (2017).
12. Sablok, G. *et al.* SeagrassDB: An open-source transcriptomics landscape for phylogenetically profiled seagrasses and aquatic plants. *Sci. Rep.* **8**, 2749 (2018).
